# Metabolites Produced by *Kaistia* sp. 32K Promote Biofilm Formation in Coculture with *Methylobacterium* sp. ME121

**DOI:** 10.3390/biology9090287

**Published:** 2020-09-13

**Authors:** Yoshiaki Usui, Tetsu Shimizu, Akira Nakamura, Masahiro Ito

**Affiliations:** 1 Graduate School of Life Sciences, Toyo University, Oura-gun, Gunma 374-0193, Japan; ysak4415@gmail.com; 2 Microbiology Research Center for Sustainability (MiCS), Faculty of Life and Environmental Sciences, University of Tsukuba, Tsukuba, Ibaraki305-8572, Japan; sakuratettyan@hotmail.com (T.S.); nakamura.akira.fm@u.tsukuba.ac.jp (A.N.); 3 Bio-Nano Electronics Research Centre, Toyo University, Kawagoe, Saitama 350-8585, Japan

**Keywords:** coculture, biofilm, Methylobacterium, *Kaistia*, motility, Methylobacterium radiotolerans, *Kaistia adipata*

## Abstract

Previously, we reported that the coculture of motile *Methylobacterium* sp. ME121 and non-motile *Kaistia* sp. 32K, isolated from the same soil sample, displayed accelerated motility of strain ME121 due to an extracellular polysaccharide (EPS) produced by strain 32K. Since EPS is a major component of biofilms, we aimed to investigate the biofilm formation in cocultures of the two strains. The extent of biofilm formation was measured by a microtiter dish assay with the dye crystal violet. A significant increase in the amount of biofilm was observed in the coculture of the two strains, as compared to that of the monocultures, which could be due to a metabolite produced by strain 32K. However, in the coculture with strain 32K, using *Escherichia coli* or *Pseudomonas aeruginosa*, there was no difference in the amount of biofilm formation as compared with the monoculture. Elevated biofilm formation was also observed in the coculture of strain ME121 with *Kaistia adipata*, which was isolated from a different soil sample. *Methylobacterium radiotolerans*, isolated from another soil sample, showed a significant increase in biofilm formation when cocultured with *K. adipata*, but not with strain 32K. We also found that the culture supernatants of strains 32K and *K. adipata* accelerated the motility of strains ME121 and *M. radiotolerans*, wherein culture supernatant of *K. adipata* significantly increased the motility of *M. radiotolerans*, as compared to that by the culture supernatant of strain 32K. These results indicated that there was a positive relationship between accelerated motility and increased biofilm formation in *Methylobacterium* spp. This is the first study to report that the metabolites from *Kaistia* spp. could specifically modulate the biofilm-forming ability of *Methylobacterium* spp. *Methylobacterium* spp. biofilms are capable of inhibiting the biofilm formation of mycobacteria, which are opportunistic pathogens that cause problems in infectious diseases. Thus, the metabolites from the culture supernatant of *Kaistia* spp. have the potential to contribute to the environment in which increased biofilm production of Methylobacterium is desired.

## 1. Introduction

*Methylobacterium* sp. ME121 and *Kaistia* sp. 32K were isolated from the same soil sample, by chance, during screening for L-glucose-assimilating bacteria, and their growth was enhanced by coculture [1,2]. *Methylobacterium* species are characterized as Gram-negative non-spore-forming bacilli, facultative methylotrophs which form pink-pigmented colonies on agar plates and have been widely observed to live on plant leaves, in soil, and in chlorinated tap water [3,4]. *Methylobacterium* species are also found in clinical samples as opportunist pathogens [4,5]. *Kaistia* species are characterized as Gram-negative non-spore-forming rod to coccus, strictly aerobic, chemoorganotrophic bacteria that form mucoid colonies on agar plates and have been observed to inhabit environments such as soil, wetland, and river sediments [6,7,8,9].

Strain ME121 is motile and has unipolar flagella, whereas strain 32K is non-motile and has no flagella [10]. In a previous study, we discovered that the motility of strain ME121 was accelerated in the coculture of strains ME121 and 32K because of an extracellular polysaccharide (EPS) produced by strain 32K [10]. The EPS derived from strain 32K was designated as K factor; it is composed of glucose and galactose in a ratio of *ca*. 1:1 and accounts for about 55% of the total weight of purified K factor. EPS is a major component of the biofilm complex [11,12]. A biofilm is a collection of microbial cells attached to a surface and surrounded by a matrix of exopolymer substances [13]. Biofilms are harmful to humans, as they cause decay of foods and are one of the causes of nosocomial infections [14]. In a medical field, such as a hospital, it is difficult to remove a biofilm formed inside an injection needle or a catheter, and this leads to nosocomial infections. In an actual medical field facing the problems, the development of a method for biofilm removal without using chemical agents, such as bactericides and antibiotics, is required, keeping in mind the health of people and resistance mechanisms of microorganisms [15]. 

Biofilm formation is closely related to bacterial motility, and its role in adhesion to solid surfaces [16,17]; during their life cycle, bacteria exist either as planktonic cells, wherein they explore the aqueous environments, or as biofilm complexes. The switch from the planktonic phase to the sessile phase involves a slowdown of metabolic activities and production of a complex mixture of the extracellular matrix, extracellular polysaccharides (EPS), proteins, and nucleic acids [18,19]. Cyclic di-GMP (c-di-GMP) is an intracellular signal transduction molecule that regulates the transition from the motile phase to the inactive phase in the bacterial cells [20]. High intracellular c-di-GMP concentrations promote biofilm formation, while low intracellular c-di-GMP levels promote motility [21]. The determinants of c-di-GMP-modulated biofilms include flagellar rotation to type IV pili contraction, EPS production, surface adhesion expression, antimicrobial resistance, and other stresses [20]. It also decreases biofilm-forming ability when motile microorganisms lose their motility [18]. Hence, we hypothesized that the biofilm-forming ability of motile microorganisms might increase with accelerated motility. Therefore, in this study, we investigated whether there was a difference in biofilm formation in monocultures and coculture of strains ME121 and 32K. In addition, we investigated whether the same effect was observed in cocultures with other *Methylobacterium* and *Kaistia* strains. 

## 2. Materials and Methods

### 2.1. Bacterial Strains and Growth Media

Bacterial strains *Methylobacterium* sp. ME121 [2], *Methylobacterium radiotolerans* [19], *Kaistia* sp. 32K [2], *Kaistia adipata* [6], *E. coli* W3110 [20], and *P. aeruginosa* PAO1 [21] were used in this study. Generally, *Methylobacterium* spp. has the chemotactic ability and also has chemotaxis-related genes [22]. *E. coli* W3110 was gifted from R. Aono. *M. radiotolerans*, *K. adipata*, and *P. aeruginosa* PAO1 were purchased from Japan Collection Microorganisms (JCM, RIKEN BioResource Center, Tsukuba, Japan). 

Strains ME121 and *M. radiotolerans* were precultured in Met medium (10.0 g of peptone, 2.0 g of yeast extract, 1.0 g of MgSO_4_, and 5 mL of methanol per liter), and strains 32K and *K. adipata* were precultured in LM medium (10.0 g of tryptone, 5.0 g of yeast extract, and 1.0 g of d-mannitol per liter). Methanol was filter-sterilized with Millex-LG filters (Millipore, pore size: 0.2 μm). *E. coli* W3110 and *P. aeruginosa* PAO1 were precultured in Miller’s LB broth (10 g of tryptone, 5 g of yeast extract, and 10 g of NaCl per liter). *E. coli* W3110 and *P. aeruginosa* PAO1 were used as the reference strains for the biofilm formation assay of strains ME121 and 32K.

d-glucose synthetic medium (Glc medium) (1.07 g of NH_4_Cl, 0.81 g of MgCl_2_, 0.75 g of KCl, 1.74 g of KH_2_PO_4_, 1.36 g of K_2_HPO_4_, 2 mL of Hutner’s trace elements, and 0.90 g of d-glucose per liter) was used for the monocultures and cocultures. Hutner’s trace elements were prepared by dissolving 22.0 g of ZnSO_4_·7H_2_O, 11.4 g of H_3_BO_3_, 5.06 g of MnCl_2_·7H_2_O, 1.16 g of CoCl_2_·5H_2_O, 1.57 g of (NH_4_)_6_Mo_7_O_24_·4H_2_O, and 1.57 g of FeSO_4_·7H_2_O in 500 mL of sterile water. EDTA·2Na (50 g) was dissolved while heating 300 mL of Milli-Q water. The pH was adjusted within the 6.5–6.8 range with KOH after the addition of each component, and the final volume was adjusted to 1 L. The solution was stored at 4 °C, for approximately two weeks, until its color changed from light green to purple-red. It was then filter-sterilized and used. 

### 2.2. Monoculture and Coculture Conditions

Strains ME121 and 32K were cultured in 10 mL of Met and LM medium, respectively (28 °C, 300 rpm, and 48 h), harvested by centrifugation (4 °C, 9100× *g*, 5 min), and then each pellet was washed with saline and suspended in 2 mL of Glc medium.

For monoculture, the cells were inoculated in Φ24 mm test tubes containing 10 mL of Glc medium to ensure an initial optical density (OD_600_) of 0.08, and the tubes were incubated at 28 °C and 300 rpm for 24 h. For the coculture, 5 mL of each bacterial suspension (OD_600_ = 0.08) was mixed in the same test tube and incubated at 28 °C and 300 rpm for 24 h.

### 2.3. Preparation of the Culture Supernatant of Strain ME121

Strain ME121 was cultured in 50 mL Met medium at 28 °C and 200 rpm for 48 h. The culture was then centrifuged (4 °C, 9100× *g*, 5 min), and the cell pellet was resuspended in 50 mL of saline and centrifuged again (4 °C, 9100× *g*, 5 min); the pellet was inoculated in a 500 mL conical flask containing 200 mL of Glc medium to achieve an initial OD_600_ of 0.08 and incubated at 28 °C and 200 rpm for 48 h. The culture supernatant was harvested by centrifugation (4 °C, 9100× *g*, 30 min), and it (200 mL) was sterilized by using a Nalgene Rapid-Flow Polyethersulfone (PES) membrane filter unit (pore diameter: 0.2 µm; Thermo Fisher Scientific, Waltham, MA, USA).

### 2.4. Preparation of Culture Supernatant of Strains 32K and K. adipata 

Strains 32K and *K. adipata* were cultured in 100 mL of LM medium at 28 °C and 200 rpm for 48 h; the cells were then centrifuged (4 °C, 9100× *g*, 5 min), and each cell culture was resuspended in 50 mL of saline and centrifuged again (4 °C, 9100× *g*, 5 min). Each cell pellet was inoculated in a 500 mL conical flask containing 200 mL of Glc medium to achieve an initial OD_600_ of 0.08 and incubated at 28 °C and 200 rpm for 48 h. The culture supernatant was harvested by centrifugation (4 °C, 9100× *g*, 30 min), and it was sterilized by using a Nalgene Rapid-Flow Polyethersulfone (PES) membrane filter unit (pore diameter: 0.2 µm; Thermo Fisher Scientific).

### 2.5. Biofilm Formation Assay

A single colony of strains ME121 and *M. radiotolerans* inoculated in Met medium, and a single colony of strains 32K and *K. adipata* inoculated in LM medium were subjected to reciprocal shaking at 28 °C and 300 rpm for 24 h. *E. coli* W3110 and *P. aeruginosa* PAO1 were subjected to reciprocal shaking at 37 °C and 200 rpm, for 16 h, in 2 mL of LB medium. Glc medium, culture supernatant of strain 32K, culture supernatant of strain ME121, and culture supernatant of *K. adipata* were each added, separately, in 5 mL batches, to a Falcon 24-well flat-bottom microplate (polystyrene, CORNING), and the culture medium was adjusted to an initial OD_600_ of 0.01. The compartment containing only the medium served as the negative control. Glc medium was used for the dilution of the supernatant. After culturing, 0.5 mL of the culture broth was removed, and 0.5 mL of 0.1% crystal violet solution was added dropwise. Growth was evaluated by measuring the OD_600_ of the culture solution. Staining of the biofilm was carried out for 30 min, under static conditions, at 25 °C. The removal of the supernatant and the dropwise addition of the solution were performed gently, to prevent any damage to the biofilm. The crystal violet solution was removed, and the biofilm was washed by adding and removing 0.75 mL of sterilized water. This process was performed three times. The microplate was placed in a 55 °C dry heater, for 10 min, to remove excess water. Then, 1 mL of 99.5% ethanol solution was added dropwise, and pipetting was performed. After leaving the plate still for 30 min, crystal violet decolorized from the biofilm. Then, Abs_570_ was measured to determine the amount of biofilm formed. The same independent experiment was performed at least more than three times. 

### 2.6. Swimming Speed of Strain ME121 and M. radiotolerans in the Culture Supernatant of Strain 32K and K. Adipata

Single colonies of strains ME121 and *M. radiotolerans* were inoculated in 10 mL of Met medium (separately) and incubated at 28 °C and 300 rpm for 48 h; the preculture broth (50 µL) was inoculated into a test tube containing 10 mL of Met medium and incubated at 28 °C and 300 rpm for 24 h. The culture broth (1 mL) was centrifuged (room temperature, 9100× *g*, 3 min), and the cells of strains ME121 and *M. radiotolerans* were resuspended in 1 mL of the swimming assay medium.

Three types of swimming assay media were tested: (i) Glc medium, (ii) culture supernatant of strain 32K, and (iii) culture supernatant of *K. adipata*. Immediately after suspension, the microbial cells were kept at 28 °C, on a glass heater; the motility of the bacteria was observed with a dark-field microscope. We recorded a video of the observed movements for about 5 s and the moving distance per second (40 frames), using a digital color camera (Leica DF310 FX). The speed of each swimming cell was calculated by using 2D movement measurement capture 2D-PTV software (Digimo, Tokyo, Japan) and analysis of the captured movie [23]. 

### 2.7. Statistical Analysis

**Figure 1:**
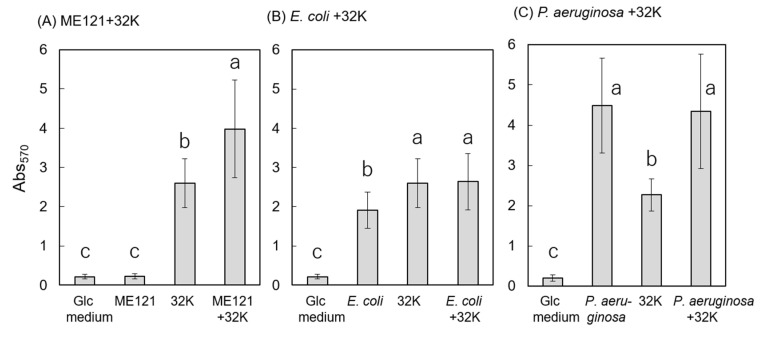
Biofilm formation during cocultivation of strain 32K with test strains**.** (**A**) Coculture of strains ME121 and 32K, (**B**) coculture of *E. coli* W3110 and strain 32K, and (**C**) coculture of *P. aeruginosa* PAO1 and strain 32K. The vertical axis is Abs_570_. Error bars are standard deviations. One-way ANOVA test was performed for equality of all means; ME121 + 32K: F_(3, 59)_ = 98.90, *p* < 0.01. *E. coli* + 32K: F_(3, 59)_ = 63.96, *p* < 0.01, *P. aeruginosa* + 32K: F_(3, 31)_ = 31.97, *p* < 0.01. Tukey’s test was performed for post hoc analysis, *p* < 0.05. Different superscript letters (a, b, c) denote significant difference from each other in all combinations.

**Figure 2:**
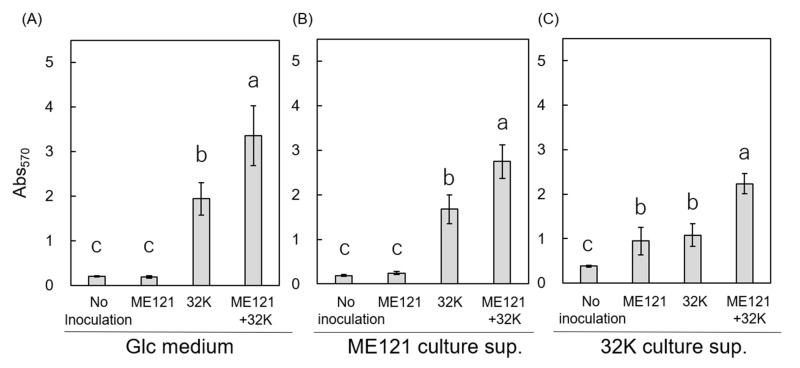
Biofilm formation of strains ME121, 32K, and ME121 + 32K in the presence of culture supernatants of strains ME121 or 32K. (**A**) Glc medium, (**B**) culture supernatant of strain ME121, and (**C**) culture supernatant of strain 32K. The vertical axis is Abs_570_, and the error bar is standard deviation. Two-way ANOVA test was performed for equality of all means; Glc medium: F_(3, 19)_ = 64.44, *p* < 0.01. ME121 culture supernatant: F_(3, 19)_ = 98.24, *p* < 0.01, 32K culture supernatant: F_(3, 19)_ = 44.65, *p* < 0.01. Tukey’s test was performed for post hoc analysis, *p* < 0.05. Different superscript letters (a, b, c) denote significant differences from each other in all combinations.

**Figure 3:**
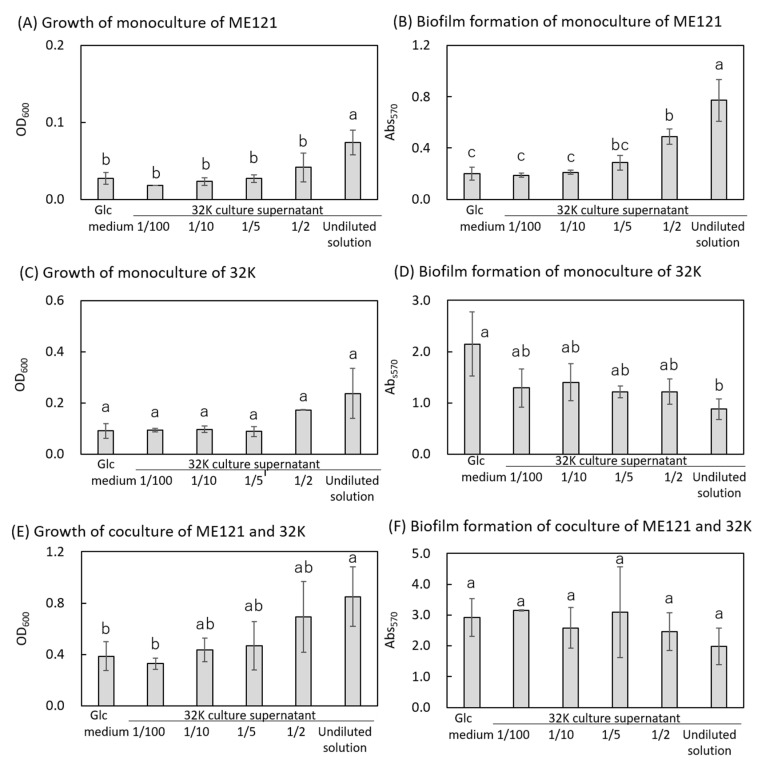
Growth and biofilm formation in monocultures of strains ME121 (**A**,**B**), 32K (**C**,**D**), coculture of strains ME121 and 32K (**E**,**F**), using culture supernatant of strain 32K diluted with Glc medium. Error bars are standard deviations. Two-way ANOVA test was performed for equality of all means; (**A**) F_(5, 21)_ = 10.75, *p* < 0.001. (**B**): F_(5, 21)_ = 23.05, *p* < 0.001, (**C**): F_(5, 21)_ = 3.66, *p* < 0.05, (**D**) F_(5, 21)_ = 2.50, *p* = 0.074, (**E**) F_(5, 21)_ = 4.33, *p* < 0.05, (**F**) F_(5, 21)_ = 0.59, *p* = 0.709. Tukey’s test was performed for post hoc analysis, *p* < 0.05. Different superscript letters (a, b, c) denote significant difference from each other in all combinations.

**Figure 4:**
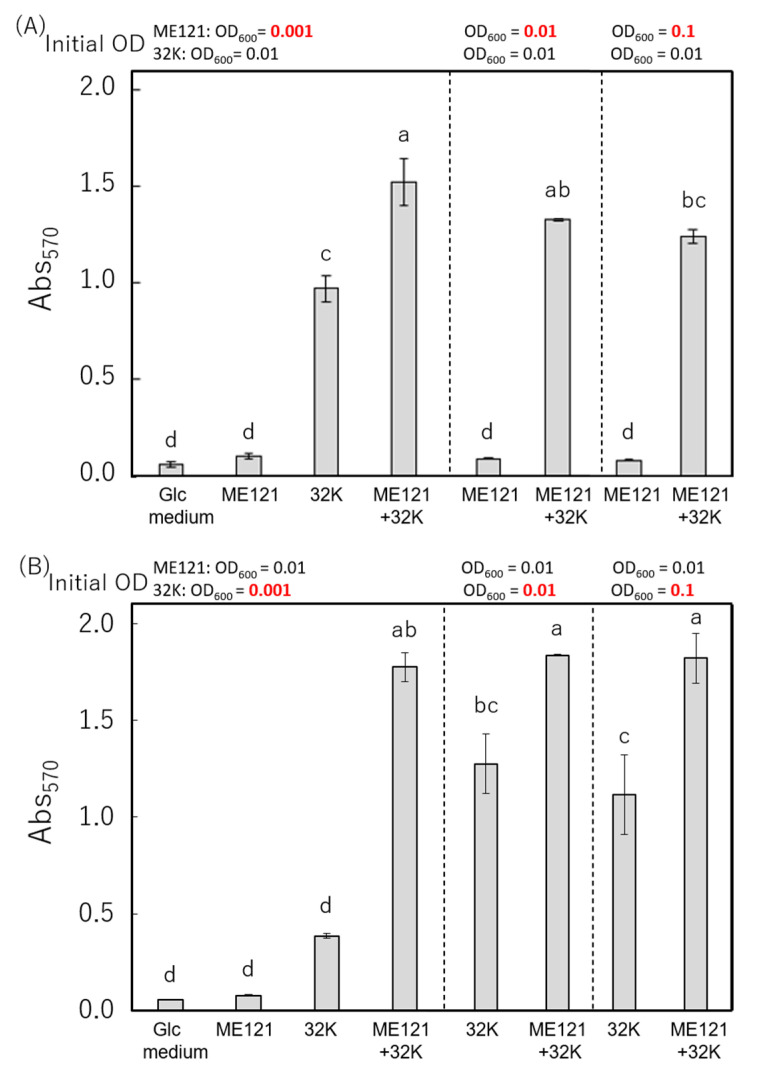
Biofilm formation in monocultures of strains ME121 and 32K, and coculture of strains ME121 and 32K when (**A**) the initial inoculum of strain ME121 was changed and (**B**) when the initial inoculum of strain 32K was changed. The vertical axis is Abs_570_, and the error bar is standard deviation. One-way ANOVA test was performed for equality of all means; (**A**): F_(7, 39)_ = 140.00, *p* < 0.001, (**B**): F_(7, 39)_ = 69.90, *p* < 0.001. Tukey’s test was performed for post hoc analysis, *p* < 0.05. Different superscript letters (a, b, c, d) denote significant differences from each other in all combinations.

**Figure 5:**
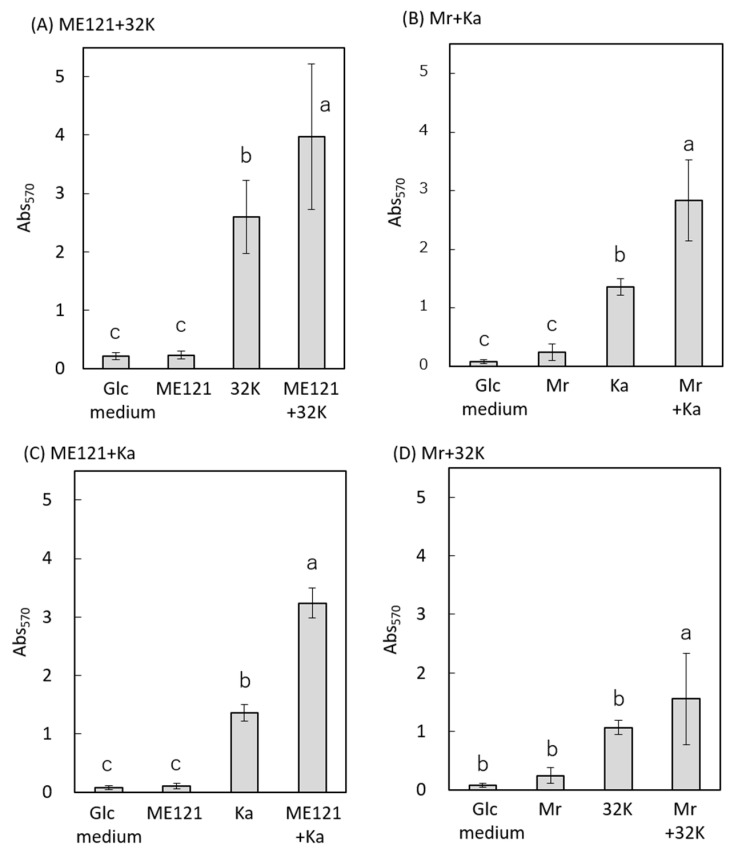
Biofilm formation during coculture of different combinations of bacterial strains. (**A**) Coculture of strains ME121 and 32K, (**B**) coculture of *M. radiotolerans* (Mr) and *K. adipata* (Ka), (**C**) coculture of strain ME121 and *K. adipata* (Ka), and (**D**) coculture of *M. radiotolerans* (Mr) and strain 32K. The vertical axis is Abs_570_, and the error bar is standard deviation. One-way ANOVA test was performed for equality of all means; (**A**): F_(3, 19)_ = 98.90, *p* < 0.001. (**B**): F_(3, 19)_ = 49.47, *p* < 0.001. (**C**): F_(3, 19)_ = 389.05, *p* < 0.001. (**D**): F_(3, 19)_ = 10.90, *p* < 0.001. Tukey’s test was performed for post hoc analysis, *p* < 0.05. Different superscript letters (a, b, c) denote significant difference from each other in all combinations.

**Figure 6:**
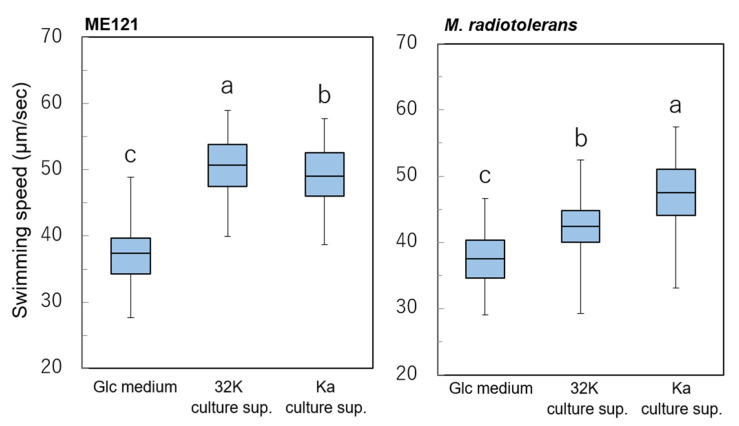
Swimming speed of strain ME121 and *M. radiotolerans* (Mr) when D-glucose synthetic medium, culture supernatant of strain 32K, and culture supernatant of *K. adipata* (Ka) were added. Half of the total values are collected in the box, and the thick line in the center of the box shows the average value. The lines that extend vertically are the remaining values, and the edges indicate the maximum and minimum values, respectively. The vertical axis shows the swimming speed (µm/s). One-way ANOVA test was performed for equality of all means; ME121: F_(2,269)_ = 225.29, *p* < 0.001. *M. radiotolerans*: F_(2,239)_ = 100.20, *p* < 0.001. Tukey’s test was performed for post hoc analysis, *p* < 0.05. Different superscript letters (a, b, c) denote significant differences from each other in all combinations.

All the results were analyzed by appropriate statistical methods (one-way analysis of variance (ANOVA), two-way ANOVA, and post hoc analysis (Tukey’s multiplex test) by the BellCurve for Excel, version 3.21, Social Survey Research Information Co., Ltd., Tokyo, Japan). Tukey test data for post hoc analysis of the results of Figure 1, Figure 2, Figure 3, Figure 4, Figure 5 and Figure 6 are listed in Supplementary Materials Tables S1–S6.

## 3. Results

### 3.1. Biofilm Formation during the Coculture of Bacterial Strains ME121 and 32K

Biofilm formation assay was performed, using Glc medium (Figure 1A). No biofilm was observed in the monoculture of strain ME121. On the other hand, biofilm was formed in the monoculture of strain 32K. A significant increase in the amount of biofilm formed was observed in the coculture of strains ME121 and 32K, as compared to that in the monoculture of strain 32K. In a similar experiment, wherein strain ME121 was replaced with *E. coli* W3110 or *P. aeruginosa* PAO1 in cocultures with strain 32K, an increase in biofilm formation was not observed (Figure 1B,C). Tukey’s multiplex test was used to test the difference between the amount of biofilm formed (mean values) in each coculture and the monocultures of their constituent strains (*p* < 0.05). A significant difference was observed between the amount of biofilm formed in the coculture of strains ME121 and 32K and the monocultures of strains ME121 and 32K, but no significant difference was observed between the control (Glc medium) and the amount of biofilm formed in the monoculture of strain ME121 (Figure 1A). Figure 1B shows that a significant difference was observed between the amount of biofilm formed in the coculture of *E. coli* and strain 32K and *E. coli* monoculture, but no significant difference was observed between the amount of biofilm formed in monoculture of strain 32K and coculture of *E. coli* and strain 32K (Figure 1B). We observed a significant difference in the amount of biofilm formed between the coculture of *P. aeruginosa* and strain 32K and monoculture of strain 32K, but not in the amount of biofilm formed between the monoculture of *P. aeruginosa* and coculture of *P. aeruginosa* and strain 32K (Figure 1C).

### 3.2. Biofilm Formation Using Culture Supernatants of Strains ME121 and 32K

Based on the results from Section 3.1, we hypothesized that strain ME121 produced a biofilm-promoting factor which promoted the biofilm formation of strain 32K or vice versa. Therefore, a biofilm formation assay was carried out by obtaining culture supernatant of strain ME121 grown in Glc medium and culture supernatant of strain 32K grown in Glc medium (Figure 2).

Tukey’s multiple comparison test was performed to assess the differences between the effects of only Glc medium and addition of the two culture supernatants on the amount of biofilm formed, after two-way ANOVA analysis. The results showed no significant difference between the amount of biofilm formed in monoculture of strain ME121 in Glc medium with no inoculum and in the presence of culture supernatant of strain ME121 (Figure 2A,B). In the case of the culture supernatant of strain 32K, there was no significant difference between the amount of biofilm formed in the monocultures of strains ME121 and 32K (Figure 2C). No significant difference was observed in all three conditions (only Glc medium, culture supernatant of strain 32K, and culture supernatant of strain ME121) when no inoculum was added. In addition, there was a significant difference between the amount of biofilm formed in monocultures of strains ME121 and 32K (except in only Glc medium and culture supernatant of strain ME121), but there was a significant difference in the amount of biofilm formed in all other combinations.

No improvement in the amount of biofilm formed by strain 32K was observed when Glc medium or culture supernatant of strain ME121 was added (Figure 2A,B). On the other hand, when the culture supernatant of strain 32K was used, the amount of biofilm formed by strain ME121 increased (Figure 2A,C). This result suggested that strain 32K produced the biofilm-promoting factor, which promoted the biofilm formation of strain ME121.

### 3.3. Relationship between Growth and Biofilm Formation during Coculture of Strains ME121 and 32K

Since the biofilm production of strain ME121 increased due to the metabolites present in the culture supernatant of strain 32K, the relationship between the growth and biofilm formation was investigated by diluting the culture supernatant of strain 32K with Glc medium (Figure 3). 

Tukey’s multiple comparison test was performed to assess the difference in proliferation between each culture (monoculture of strain ME121, monoculture of strain 32K, and coculture of strains ME121 and 32K) after addition of different dilutions of culture supernatant of strain 32K. No significant difference in the growth was observed between the monocultures of strains ME121 and 32K in only Glc medium, 100-fold, 10-fold, 5-fold, and 2-fold diluted culture supernatant of strain 32K. Moreover, there was no significant difference between the amount of biofilm formed in the monoculture of strain 32K and the coculture of strains ME121 and 32K at 100-fold dilution of the culture supernatant. All other combinations were significantly different (Figure 3A,C,E). 

The effects of the dilutions of culture supernatant of strain 32K on the amount of biofilm formed were also investigated for monoculture of strains ME121 and 32K and their coculture. We observed a significant difference in the ratio of the amount of biofilm formed in all dilutions of culture supernatant of strain 32K (Figure 3B,D,F). Two-way ANOVA analysis was used to evaluate whether there is a relationship between bacterial growth and biofilm formation. The analysis showed that the difference between the amount of biofilm formed in the monoculture of strain ME121 and the coculture was significant under all dilutions of culture supernatant of strain 32K. There was a significant difference in the amount of biofilm formed between monoculture of strain 32K and the coculture (except in case of only Glc medium and 10-fold dilution), and monocultures of strains ME121 and 32K in only Glc medium. No significant difference was observed in the other combinations (Figure 3B,D,F).

The growth and amount of biofilm formed in monoculture of strain ME121 improved when culture supernatant of strain 32K was added (Figure 3A,B). On the other hand, in the case of monoculture of strain 32K, its culture supernatant showed no significant difference in improving its growth, but it decreased the amount of biofilm formed (Figure 3C,D). Growth of the strain also improved when strains ME121 and 32K were cocultured, but the amount of the biofilm formed was not significantly different (Figure 3E,F). It has been reported that *Pseudomonas aeruginosa* produces an EPS-degrading enzyme in a nutrient-starved state and uses the degraded polysaccharide as a nutrient source (32). After the process of biofilm formation is completed, the biofilm is decomposed, and bacteria are eliminated according to the surrounding nutritional conditions and stress environment (23, 24, 26). Therefore, in our study, after 48 h of cocultivation, many cells continued to form the biofilm, and the number of planktonic bacteria was not large. However, in the culture supernatant of strain 32K, the cells that originally formed biofilm were detached from the biofilm after 48 h, and the remaining cells existed as planktonic bacteria. As a result, even when only strain 32K was cultured or strains ME121 and 32K were cocultured, the cells in the biofilm obtained nutrients from the aged biofilm and were released from the biofilm in search of a new desirable environment. It was speculated that the amount of biofilm formed in the supernatant was reduced or unchanged. Another possibility is that the metabolites contained in the culture supernatant of strain 32K regulated the amount of biofilm formed under each culture condition.

### 3.4. Comparison of the Amount of Biofilm Formed due to Difference in Initial Inoculum Size

There is a specific relationship between dominant and rare microbial species in their natural habitats. Therefore, we investigated the effect of different initial inoculum size of strains ME121 and 32K during biofilm formation. Tukey’s multiplex test was performed to assess the difference between the amount of biofilm formed in each coculture (mean values) when the initial inoculum of the bacterial strains was varied (*p* < 0.05). The results are shown in Figure 4.

When the initial inoculum of strain ME121 was reduced to 1/10 or increased to 10 times the original inoculum, it did not affect biofilm formation during coculture with strain 32K (Figure 4A). On the other hand, when the initial inoculum of strain 32K was reduced 1/10-fold, the amount of biofilm formed in the monoculture of strain 32K decreased, but the amount of biofilm formed was the same in coculture with strain ME121 when the initial inoculum of strain 32K was varied (Figure 4B). We found that the viable cell count of strains ME121 and 32K at OD_600_ of 0.01 was 6.15 × 10^6^ CFU/mL and 1.31 × 10^7^ CFU/mL, respectively. It was speculated that, when the bacterial cell number of strain 32K exceeds a certain threshold value, the production of EPS and other compounds starts, and a large amount of biofilm can be formed in association with strain ME121.

### 3.5. Comparison of Biofilm Formation during Coculture with Another Methylobacterium and Kaistia Strain

A significant increase in the amount of biofilm formed was observed when strains ME121 and 32K were cocultured. We examined whether this phenomenon occurred even when the strains were cocultured with another *Methylobacterium* or *Kaistia* strain. *M. radiotolerans* (hereafter referred to as Mr) and *K. adipata* (hereafter referred to as Ka) were the two other strains used for this experiment. [6,19]. In this study, we used Mr and Ka as representative *Methylobacterium* and *Kaistia* bacteria from different isolation sites. Tukey’s multiplex test was performed to test the difference between the biofilm formed (mean values) in each coculture (*p* < 0.05). The results are shown in Figure 5.

An increase in the amount of biofilm formed was also observed during coculture of strains Mr and Ka compared to monoculture of Ka (Figure 5B). In addition, an increase was also seen during coculture of strains ME121 and Ka compared to monoculture of Ka (Figure 5C). However, no significant increase in the amount of biofilm formed was observed in coculture of strains Mr and 32K compared to monoculture of 32K (Figure 5D). Thus, we concluded that the amount of biofilm increased even in the coculture of strain ME121 and *Kaistia* spp. bacteria that were isolated from different soil samples.

### 3.6. Comparison of Swimming Speed during Coculture with Another Methylobacterium and Kaistia Bacteria

Previously, we reported that the swimming speed of strain ME121 increases when culture supernatant of strain 32K is added. As a mechanism for accelerating the swimming of strain ME121, it was found that the flagellar motor of strain ME121 had a simultaneous increase in motor driving force when exposed to the K factor contained in the 32K culture supernatant [10]. It is known that the flagellar motor stator constantly exchanges its number of units in a motor [24]. In this way, the torque output of the flagellar motor is adjusted by the number of stator units. When strain ME121 is also exposed to the K factor, the number of stators taken into the motor increases, and we believe that the swimming speed increases due to the increase in motor torque. Therefore, swimming speeds of strains ME121 and Mr were measured when Glc medium, culture supernatant of strain 32K, and culture supernatant of strain Ka were added (Supplementary Materials Videos S1 and S2). Tukey’s multiplex test was performed to test the difference between the swimming speeds (mean values) of strains ME121 and Mr in the presence of culture supernatant of strain ME121 or strain Ka (*p* < 0.05). The results are shown in Figure 6. Characteristically, we observed that both strains ME121 and Mr had accelerated swimming speeds, regardless of the culture supernatant used. Culture supernatant of strain 32K accelerated the swimming speed of strain ME121 more than that by the culture supernatant of strain Ka. On the other hand, significant acceleration in the swimming speed of strain Mr was observed when culture supernatant of strain Ka was added. 

The cocultivation of strains 32K and Mr did not contribute to the increase in the amount of biofilm formed, as compared to that of the cocultivation of strains Ka and Mr (Figure 5D). Similarly, in terms of swimming speed, the acceleration of swimming speed with culture supernatant of strain 32K was lower, as compared to that with the culture supernatant of strain Ka. Figure 5 and Figure 6 suggest that the biofilm formation and acceleration of swimming speed are positively correlated between *Methylobacterium* spp. and the *Kaistia* spp.

**Figure 7:**
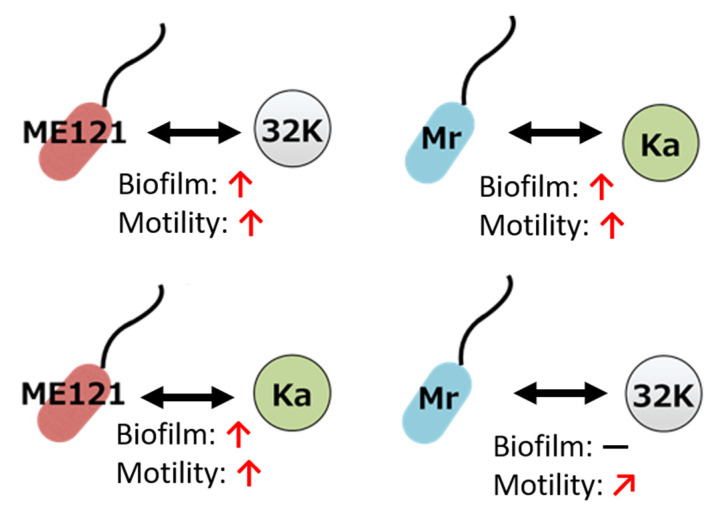
Schematic diagram of the effects on biofilm formation and motility of each strain. “**↑**” shows increased biofilm formation and accelerated motility. “**－**” shows no effect on biofilm formation. “**↗**” shows moderately accelerated motility.

A schematic diagram of the effect of different cocultures on biofilm formation and motility is shown in Figure 7. Except for the coculture of strains *Mr* and 32K, increased biofilm formation and accelerated motility was observed in all other combinations of cocultures.

## 4. Discussion

### Relationship between Motility Acceleration of Strain ME121 and Biofilm Formation 

**Figure 8:**
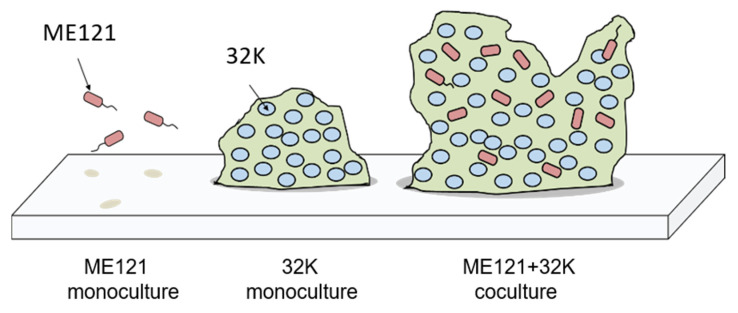
Schematic representation of biofilm formation of strains ME121 and 32K. In the synthetic medium used in this experiment, strain ME121 does not form a biofilm when cultured alone, whereas monoculture of strain 32K forms a biofilm. It was speculated that a larger biofilm was formed in the coculture of strains ME121 and 32K.

After strain ME121 recognizes the K factor produced by strain 32K, the rotational force of the flagellar motor of strain ME121 is accelerated [10]. The advantages of motility acceleration for strain ME121 are speculated to be faster access to the biofilm of strain 32K and enhanced invasion into the strain 32K biofilm complex. We elucidated that the growth of strain ME121 also improved due to the growth enhancer produced by strain 32K (Figure 3). Strain ME121 may also be capable of producing a strain 32K-growth-improving factor and -biofilm-promoting factor in a coculture with strain 32K. Strain 32K forms biofilms in a monoculture, but in a coculture with strain ME121, it enhances biofilm production. Therefore, it is considered that both strains ME121 and 32K affect each other, and a larger biofilm can be formed in their cocultures (Figure 8). It is conceivable that strains ME121 and 32K grow in a symbiotic relationship, such as coevolution in nature. Future prospects of the study include observation of the distribution and abundance ratios of the two strains and the biofilm formation process over time in the coculture of strains ME121 and 32K.

The amount of biofilm formed was significantly increased in the coculture of strains ME121 and 32K, and strains ME121 and Ka. However, there was no increase in the amount of biofilm formed in the coculture of strains *Mr* and 32K (Figure 5 and Figure 7). In addition, the motility of strain Mr was moderately accelerated in the swimming assay when the culture supernatant of strain 32K was added (Figure 6 and Figure 7). Strains ME121 and 32K were isolated from the same soil [2], whereas strain Mr was isolated from brown rice in Japan [19], and strain Ka was isolated from soil in Korea [6]. This suggests that increased biofilm formation and acceleration of motility in *Methylobacterium* spp. and *Kaistia* spp. depend on the combination of the bacterial strains and not on the source of the bacterium.

Since a biofilm is more resistant to various stress conditions than the planktonic cells, it was postulated that strains ME121 and 32K both grow to form a biofilm to survive in nature. It was considered that the metabolites produced by strain 32K cause an increase in the growth and motility of strain ME121 and vice versa. In the future, it is expected that the specific factors involved in the process will be identified by conducting more cocultivation tests of *Methylobacterium* spp. and *Kaistia* spp., using accelerated motility and improvement in biofilm formation as indicators.

In nature, microorganisms do not coexist with the same cell number for each species, but they exist as dominant and rare species in a habitat. Thus, we varied the initial inoculum size to investigate the effects of inoculum size on the biofilm formation of strains ME121 and 32K. As a result, even when the initial OD of strain 32K was reduced to OD_600_ of 0.001, the amount of biofilm increased significantly during the coculture (Figure 4). The results showed that when the initial inoculum of strain 32K exceeded a certain threshold value, the production of EPS and other molecules began, and a large biofilm was formed together with strain ME121. We also observed that even a small number of strain 32K cells could interact with the cells of strain ME121 to form a large biofilm.

We previously reported that the accelerated motility of strain ME121 cocultured with strain 32K was related to EPS produced by strain 32K [10]. EPS is the main component of a biofilm. During biofilm formation, information is exchanged between cells, using quorum sensing and autoinducers [25,26]. Acyl homoserine lactone compounds are used as common quorum sensing substances in Gram-negative bacteria [26,27]. It is inferred that, in the current study also, information was exchanged by these substances during the biofilm formation of the coculture of strains ME121 and 32K. As the genome sequence of strain ME121 has been elucidated [2], in the future, we will search for genes related to quorum sensing and quantify the expression levels of those genes, using monoculture of strain ME121 and its coculture with strain 32K, using real-time PCR, qPCR, etc. By analyzing not only the genes involved in quorum sensing but also the genes related to polysaccharide production and quantifying the expression of these genes, biofilm formation during coculture of strains ME121 and 32K can be analyzed at the expression level. This is expected to lead to the elucidation of the mechanism of biofilm formation.

Through a series of studies, we would like to discuss new findings in the symbiotic relationship between *Methylobacterium* spp. and *Kaistia* spp. and the possibility of controlling bacterial biofilm formation. *Methylobacterium* spp. biofilms are known to inhibit mycobacterial biofilm formation, which causes hospital-related infection problems [28]. Based on the results of this study, it is expected that the metabolites contained in the culture supernatant of *Kaistia* spp. can be used to increase the production of *Methylobacterium* spp. biofilms as a biological material, and to modulate the amount of metabolites of *Methylobacterium* spp. biofilm as a microbial material. 

It is expected that the investigation of the relationship between biofilms formed by complex microbial systems and their motility can be applied to various fields, such as drug discovery [29]. If future studies can elucidate the mechanism of motility improvement and identify the motility improvement factor, then biofilm formation can be modulated artificially.

## Supplementary Materials

The following data are available online at https://www.mdpi.com/2079-7737/9/9/287/s1. Video S1: Swimming motility of ME121 with Glc medium, 32K supernatant and *K. adipata* (Ka) supernatant. Video S2: Swimming motility of *M. radiotolerans* with Glc medium, 32K supernatant and *K*. *adipata* (Ka) supernatant. Table S1: Tukey test data for post hoc analysis of the results in Figure 1. Table S2: Tukey test data for post hoc analysis of the results in Figure 2. Table S3: Tukey test data for post hoc analysis of the results in Figure 3. Table S4: Tukey test data for post hoc analysis of the results in Figure 4. Table S5: Tukey test data for post hoc analysis of the results in Figure 5. Table S6: Tukey test data for post hoc analysis of the results in Figure 6.
