# Supplementary material for: Metabolites Produced by Kaistia sp. 32K Promote Biofilm Formation in Coculture with Methylobacterium sp. ME121
Source: Biology (Basel). 2020 Sep 13;9(9):287. doi: 10.3390/biology9090287 (PMC7563137; doi:10.3390/biology9090287)
Supplement: Supplementary file 1 [file biology-09-00287-s001.zip › biology-902103-supplementary-final/supplimentary files/biology-902103-Supplementary materials.docx]

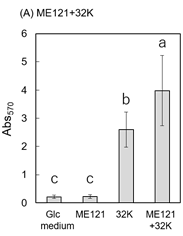
**Table S1.** Tukey test data for post hoc analysis of the results in Figure 1.

| **Figure 1** | | | | | | | | | | | | | | | | |
| --- | --- | --- | --- | --- | --- | --- | --- | --- | --- | --- | --- | --- | --- | --- | --- | --- |
| Multiple comparison analysis testing | | | | | |  |  |  |  |  |  |  |  |  |  |  |
| Figure 1A | Coculture of strains ME121 and 32K | | | | | | |  |  |  |  |  |  |  |  |  |
| Method | Level 1 | Level 2 | Average 1 | Average 2 | Difference | Standard deviation | Statistics | P value | *：P<0.05 **：P<0.01 | Figure 1A | Ave. | ME121+32K | 32K | ME121 | Glc medium | Symbol |
| Tukey | Glc medium | ME121 | 0.2143 | 0.2297 | 0.0154 | 0.2641 | 0.0583 | 0.9999 |  | ME121+32K | 3.9767 | a | ― | ― | ― | a |
|  | Glc medium | 32K | 0.2143 | 2.5993 | 2.3850 | 0.2641 | 9.0301 | ####### | ** | 32K | 2.5993 | ＊ | b | ― | ― | b |
|  | Glc medium | ME121 +32K | 0.2143 | 3.9767 | 3.7623 | 0.2641 | 14.2449 | ####### | ** | ME121 | 0.2297 | ＊ | ＊ | c | ― | c |
|  | ME121 | 32K | 0.2297 | 2.5993 | 2.3696 | 0.2641 | 8.9718 | ####### | ** | Glc medium | 0.2143 | ＊ | ＊ | c | c | c |
|  | ME121 | ME121 +32K | 0.2297 | 3.9767 | 3.7469 | 0.2641 | 14.1866 | ####### | ** | **Number of asterisks** |  | 3 | 2 | 0 | 0 |  |
|  | 32K | ME121 +32K | 2.5993 | 3.9767 | 1.3773 | 0.2641 | 5.2149 | ####### | ** | The symbols with a, b, and c are assigned from the one with the largest number of asterisks. | | | | | | |
|  |  |  |  |  |  |  |  |  |  |  |  |  |  |  |  |  |
| Figure 1B | Coculture of *E. coli* W3110 and strain 32K | | | | |  |  |  |  |  |  |  |  |  |  |  |
| Method | Level 1 | Level 2 | Ave. 1 | Ave. 2 | Difference | Standard deviation | Statistics | P value | *：P<0.05 **：P<0.01 | Figure 1B | Ave. | W3110+32K | 32K | W3110 | Glc medium | Symbol |
| Tukey | Glc medium | W3110 | 0.2143 | 1.9140 | 1.6997 | 0.2006 | 8.4715 | ####### | ** | W3110+32K | 2.6380 | a | ― | ― | ― | a |
|  | Glc medium | 32K | 0.2143 | 2.5993 | 2.3850 | 0.2006 | 11.8873 | ####### | ** | 32K | 2.5993 | a | a | ― | ― | 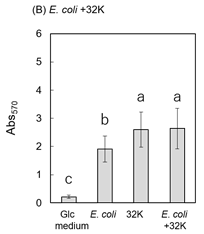a |
|  | Glc medium | W3110 +32K | 0.2143 | 2.6380 | 2.4237 | 0.2006 | 12.0801 | ####### | ** | W3110 | 1.9140 | ＊ | ＊ | b | ― | b |
|  | W3110 | 32K | 1.9140 | 2.5993 | 0.6853 | 0.2006 | 3.4158 | 0.0064 | ** | Glc medium | 0.2143 | ＊ | ＊ | ＊ | c | c |
|  | W3110 | W3110 +32K | 1.9140 | 2.6380 | 0.7240 | 0.2006 | 3.6086 | 0.0036 | ** | **Number of asterisks** |  | 2 | 2 | 1 | 0 |  |
|  | 32K | W3110 +32K | 2.5993 | 2.6380 | 0.0387 | 0.2006 | 0.1927 | 0.9974 |  |  |  |  |  |  |  |  |
|  |  |  |  |  |  |  |  |  |  |  |  |  |  |  |  |  |
| Figure 1C | Coculture of *P. aeruginosa* PAO1 and strain 32K | | | | | | | |  | Figure 1C | Ave. | Pa | Pa+32K | 32K | Glc medium | Symbol |
| Method | Level 1 | Level 2 | Ave. 1 | Ave. 2 | Difference | Standard deviation | Statistics | P value | *：P<0.05 **：P<0.01 | Pa | 4.4863 | a | ― | ― | ― | 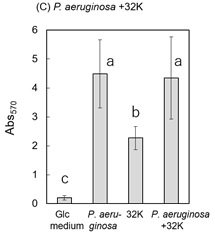a |
| Tukey | Glc medium | Pa | 0.2070 | 4.4863 | 4.2793 | 0.5048 | 8.4765 | ####### | ** | Pa+32K | 4.3413 | a | a | ― | ― | a |
|  | Glc medium | 32K | 0.2070 | 2.2675 | 2.0605 | 0.5048 | 4.0815 | 0.0018 | ** | 32K | 2.2675 | ＊ | ＊ | b | ― | b |
|  | Glc medium | Pa +32K | 0.2070 | 4.3413 | 4.1343 | 0.5048 | 8.1893 | ####### | ** | Glc medium | 0.2070 | ＊ | ＊ | ＊ | c | c |
|  | Pa | 32K | 4.4863 | 2.2675 | 2.2188 | 0.5048 | 4.3950 | ####### | ** | **Number of asterisks** |  | 2 | 2 | 1 | 0 |  |
|  | Pa | Pa +32K | 4.4863 | 4.3413 | 0.1450 | 0.5048 | 0.2872 | 0.9916 |  |  |  |  |  |  |  |  |
|  | 32K | Pa +32K | 2.2675 | 4.3413 | 2.0738 | 0.5048 | 4.1078 | 0.0017 | ** |  |  |  |  |  |  |  |

**Table S2.** Tukey test data for post hoc analysis of the results in Figure 2.

| **Figure 2** | | | | | | | | | | |
| --- | --- | --- | --- | --- | --- | --- | --- | --- | --- | --- |
| Multiple comparison test of “factor (combination)” at each level of “factor (medium)” | | | | | | |  |  |  |  |
| Method | Factor (Medium) | Level 1 | Level 2 | Average 1 | Average 2 | Difference | Standard deviation | Statistics | P value | *：P<0.05 **：P<0.01 |
| Tukey | Glc medium | No incubation | ME121 | 0.2044 | 0.1904 | 0.0140 | 0.2086 | 0.0671 | 0.9999 |  |
|  |  | No incubation | 32K | 0.2044 | 1.9400 | 1.7356 | 0.2086 | 8.3195 | P < 0.001 | ** |
|  |  | No incubation | ME121+32K | 0.2044 | 3.3560 | 3.1516 | 0.2086 | 15.1070 | P < 0.001 | ** |
|  |  | ME121 | 32K | 0.1904 | 1.9400 | 1.7496 | 0.2086 | 8.3866 | P < 0.001 | ** |
|  |  | ME121 | ME121+32K | 0.1904 | 3.3560 | 3.1656 | 0.2086 | 15.1741 | P < 0.001 | ** |
|  |  | 32K | ME121+32K | 1.9400 | 3.3560 | 1.4160 | 0.2086 | 6.7875 | P < 0.001 | ** |
|  | ME121 supernatant | No incubation | ME121 | 0.1880 | 0.2418 | 0.0538 | 0.2086 | 0.2579 | 0.9939 |  |
|  |  | No incubation | 32K | 0.1880 | 1.6740 | 1.4860 | 0.2086 | 7.1231 | P < 0.001 | ** |
|  |  | No incubation | ME121+32K | 0.1880 | 2.7480 | 2.5600 | 0.2086 | 12.2712 | P < 0.001 | ** |
|  |  | ME121 | 32K | 0.2418 | 1.6740 | 1.4322 | 0.2086 | 6.8652 | P < 0.001 | ** |
|  |  | ME121 | ME121+32K | 0.2418 | 2.7480 | 2.5062 | 0.2086 | 12.0133 | P < 0.001 | ** |
|  |  | 32K | ME121+32K | 1.6740 | 2.7480 | 1.0740 | 0.2086 | 5.1482 | P < 0.001 | ** |
|  | 32K supernatant | No incubation | ME121 | 0.3786 | 0.9434 | 0.5648 | 0.2086 | 2.7073 | 0.0448 | * |
|  |  | No incubation | 32K | 0.3786 | 1.0786 | 0.7000 | 0.2086 | 3.3554 | 0.0082 | ** |
|  |  | No incubation | ME121+32K | 0.3786 | 2.2326 | 1.8540 | 0.2086 | 8.8870 | P < 0.001 | ** |
|  |  | ME121 | 32K | 0.9434 | 1.0786 | 0.1352 | 0.2086 | 0.6481 | 0.9157 |  |
|  |  | ME121 | ME121+32K | 0.9434 | 2.2326 | 1.2892 | 0.2086 | 6.1797 | P < 0.001 | ** |
|  |  | 32K | ME121+32 | 1.0786 | 2.2326 | 1.1540 | 0.2086 | 5.5316 | P < 0.001 | ** |


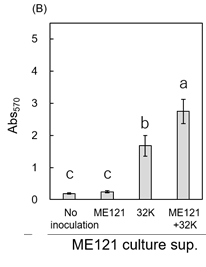

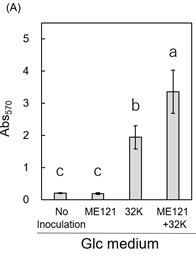


| **(A) Glc medium** | **Ave.** | **121+32K** | **32K** | **ME121** | **No inoculation** | **Symbol** |
| --- | --- | --- | --- | --- | --- | --- |
| ME121+32K | 3.3560 | a | ― | ― | ― | a |
| 32K | 1.9400 | ＊ | b | ― | ― | b |
| No inoculation | 0.2044 | ＊ | ＊ | c | ― | c |
| ME121 | 0.1904 | ＊ | ＊ | c | c | c |
| **Number of asterisks** |  | 3 | 2 | 0 | 0 |  |
| The symbols with a, b, and c are assigned from the one with the largest number of asterisks. | | | | | | |
|  |  |  |  |  |  |  |
| (B) ME121 supernatant | Ave. | 121+32K | 32K | ME121 | No inoculation | Symbol |
| ME121+32K | 2.7480 | a | ― | ― | ― | a |
| 32K | 1.6740 | ＊ | b | ― | ― | b |
| ME121 | 0.2418 | ＊ | ＊ | c | ― | c |
| No inoculation | 0.1880 | ＊ | ＊ | c | c | c |
| **Number of asterisks** |  | 3 | 2 | 0 | 0 |  |
|  |  |  |  |  |  |  |
| (C) 32K supernatant | Ave. | 121+32K | 32K | ME121 | No inoculation | Symbol |
| ME121+32K | 2.2326 | a | ― | ― | ― | a |
| 32K | 1.0786 | ＊ | b | ― | ― | b |
| ME121 | 0.9434 | ＊ | b | b | ― | b |
| No inoculation | 0.3786 | ＊ | ＊ | ＊ | c | c |
| **Number of asterisks** |  | 3 | 1 | 1 | 0 |  |


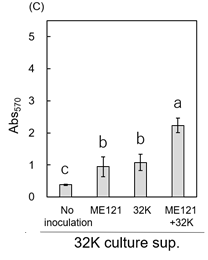


**Table S3.** Tukey test data for post hoc analysis of the results in Figure 3.

| **Figure 3** | | | | | | | | | | | | | | | | | | | | |
| --- | --- | --- | --- | --- | --- | --- | --- | --- | --- | --- | --- | --- | --- | --- | --- | --- | --- | --- | --- | --- |
| Multiple comparison analysis testing | | | | |  |  |  |  |  |  |  |  |  |  |  |  |  |  |  |  |
| (A) Growth of monoculture of ME121 | | | | |  |  |  |  |  |  |  |  |  |  |  |  |  |  |  |  |
| Method | Level 1 | Level 2 | Average 1 | Average 2 | Difference | Standard deviation | Statistics | P value | *：P<0.05 **：P<0.01 | | | (A) | Ave. | 32K sup | 1/2 | 1/5 | Glc medium | 1/10 | 1/100 | Symbol |
| Tukey | Glc medium | 1/100 | 0.0272 | 0.0167 | 0.0105 | 0.0096 | 1.0961 | 0.8759 |  |  |  | 32K sup | 0.0736 | a | ― | ― | ― | ― | ― | a |
|  | Glc medium | 1/10 | 0.0272 | 0.0233 | 0.0039 | 0.0096 | 0.4024 | 0.9984 |  |  |  | 1/2 | 0.0413 | ＊ | b | ― | ― | ― | ― | b |
|  | Glc medium | 1/5 | 0.0272 | 0.0273 | 0.0001 | 0.0096 | 0.0139 | 1.0000 |  |  |  | 1/5 | 0.0273 | ＊ | b | b | ― | ― | ― | b |
|  | Glc medium | 1/2 | 0.0272 | 0.0413 | 0.0141 | 0.0096 | 1.4707 | 0.6862 |  |  |  | Glc medium | 0.0272 | ＊ | b | b | b | ― | ― | b |
|  | Glc medium | 32K supernatant | 0.0272 | 0.0736 | 0.0464 | 0.0083 | 5.5751 | P < 0.001 | ** |  |  | 1/10 | 0.0233 | ＊ | b | b | b | b | ― | b |
|  | 1/100 | 1/10 | 0.0167 | 0.0233 | 0.0067 | 0.0107 | 0.6205 | 0.9878 |  |  |  | 1/100 | 0.0167 | ＊ | b | b | b | b | b | b |
|  | 1/100 | 1/5 | 0.0167 | 0.0273 | 0.0107 | 0.0107 | 0.9928 | 0.9137 |  |  |  | **Number of asterisks** |  | 5 | 0 | 0 | 0 | 0 | 0 |  |
|  | 1/100 | 1/2 | 0.0167 | 0.0413 | 0.0247 | 0.0107 | 2.2957 | 0.2513 |  |  |  | The symbols with a and b are assigned from the one with the largest number of asterisks. | | | | | | | | |
|  | 1/100 | 32K supernatant | 0.0167 | 0.0736 | 0.0569 | 0.0096 | 5.9243 | P < 0.001 | ** |  |  | 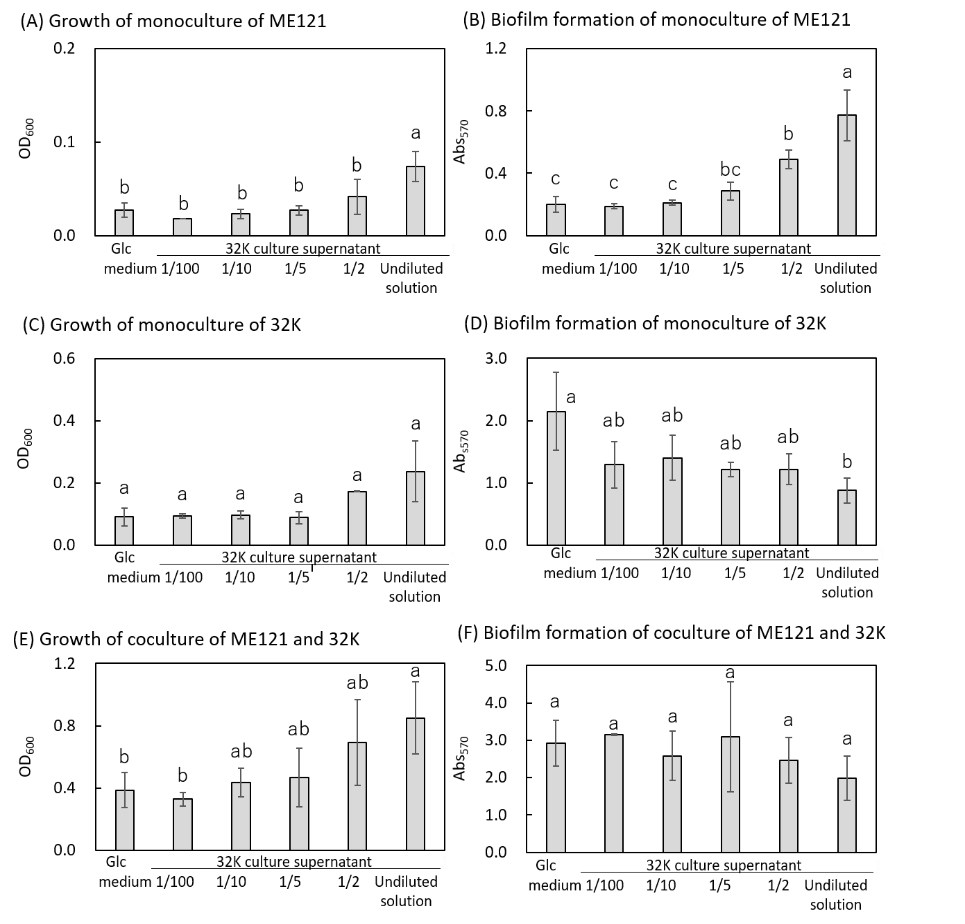 | | | | | | | | |
|  | 1/10 | 1/5 | 0.0233 | 0.0273 | 0.0040 | 0.0107 | 0.3723 | 0.9989 |  |  |  |  |  |  |  |  |  |  |  |  |
|  | 1/10 | 1/2 | 0.0233 | 0.0413 | 0.0180 | 0.0107 | 1.6753 | 0.5653 |  |  |  |  |  |  |  |  |  |  |  |  |
|  | 1/10 | 32K supernatant | 0.0233 | 0.0736 | 0.0503 | 0.0096 | 5.2306 | P < 0.001 | ** |  |  |  |  |  |  |  |  |  |  |  |
|  | 1/5 | 1/2 | 0.0273 | 0.0413 | 0.0140 | 0.0107 | 1.3030 | 0.7794 |  |  |  |  |  |  |  |  |  |  |  |  |
|  | 1/5 | 32K  supernatant | 0.0273 | 0.0736 | 0.0463 | 0.0096 | 4.8143 | 0.0022 | ** |  |  |  |  |  |  |  |  |  |  |  |
|  | 1/2 | 32K  supernatant | 0.0413 | 0.0736 | 0.0323 | 0.0096 | 3.3576 | 0.0386 | * |  |  |  |  |  |  |  |  |  |  |  |
|  |  |  |  |  |  |  |  |  |  |  |  |  |  |  |  |  |  |  |  |  |
| (B) Biofilm formation of monoculture of ME121 | | | | | |  |  |  |  |  |  |  |  |  |  |  |  |  |  |  |
| Method | Level 1 | Level 2 | Average 1 | Average 2 | Difference | Standard deviation | Statistics | P value | *：P<0.05 **：P<0.01 | | | (B) | Ave. | 32K sup | 1/2 | 1/5 | Glc medium | 1/10 | 1/100 | Symbol |
| Tukey | Glc medium | 1/100 | 0.1996 | 0.1893 | 0.0103 | 0.0743 | 0.1382 | 1.0000 |  |  |  | 32K sup | 0.7718 | a | ― | ― | ― | ― | ― | a |
|  | Glc medium | 1/10 | 0.1996 | 0.2087 | 0.0091 | 0.0743 | 0.1221 | 1.0000 |  |  |  | 1/2 | 0.4887 | ＊ | b | ― | ― | ― | ― | b |
|  | Glc medium | 1/5 | 0.1996 | 0.2817 | 0.0821 | 0.0743 | 1.1050 | 0.8723 |  |  |  | 1/5 | 0.2817 | ＊ | b | c | ― | ― | ― | bc |
|  | Glc medium | 1/2 | 0.1996 | 0.4887 | 0.2891 | 0.0743 | 3.8921 | 0.0136 | * |  |  | Glc medium | 0.2087 | ＊ | ＊ | c | c | ― | ― | c |
|  | Glc medium | 32K  supernatant | 0.1996 | 0.7718 | 0.5722 | 0.0643 | 8.8962 | P < 0.001 | ** |  |  | 1/10 | 0.1996 | ＊ | ＊ | c | c | c | ― | c |
|  | 1/100 | 1/10 | 0.1893 | 0.2087 | 0.0193 | 0.0830 | 0.2328 | 0.9999 |  |  |  | 1/100 | 0.1893 | ＊ | ＊ | c | c | c | c | c |
|  | 1/100 | 1/5 | 0.1893 | 0.2817 | 0.0923 | 0.0830 | 1.1120 | 0.8694 |  |  |  | **Number of asterisks** |  | 5 | 3 | 0 | 0 | 0 | 0 |  |
|  | 1/100 | 1/2 | 0.1893 | 0.4887 | 0.2993 | 0.0830 | 3.6049 | 0.0239 | * |  |  | The symbols with a, b and c are assigned from the one with the largest number of asterisks. | | | | | | | | |
|  | 1/100 | 32K supernatant | 0.1893 | 0.7718 | 0.5825 | 0.0743 | 7.8426 | P < 0.001 | ** |  |  |  |  |  |  |  |  |  |  |  |
|  | 1/10 | 1/5 | 0.2087 | 0.2817 | 0.0730 | 0.0830 | 0.8791 | 0.9462 |  |  |  |  |  |  |  |  |  |  |  |  |
|  | 1/10 | 1/2 | 0.2087 | 0.4887 | 0.2800 | 0.0830 | 3.3720 | 0.0376 | * |  |  |  |  |  |  |  |  |  |  |  |
|  | 1/10 | 32K  supernatant | 0.2087 | 0.7718 | 0.5631 | 0.0743 | 7.5823 | P < 0.001 | ** |  |  |  |  |  |  |  |  |  |  |  |
|  | 1/5 | 1/2 | 0.2817 | 0.4887 | 0.2070 | 0.0830 | 2.4929 | 0.1838 |  |  |  |  |  |  |  |  |  |  |  |  |
|  | 1/5 | 32K  supernatant | 0.2817 | 0.7718 | 0.4901 | 0.0743 | 6.5994 | P < 0.001 | ** |  |  |  |  |  |  |  |  |  |  |  |
|  | 1/2 | 32K  supernatant | 0.4887 | 0.7718 | 0.2831 | 0.0743 | 3.8122 | 0.0159 | * |  |  |  |  |  |  |  |  |  |  |  |
|  |  |  |  |  |  |  |  |  |  |  |  |  |  |  |  |  |  |  |  |  |
| (C) Growth of monoculture of 32K | | | | |  |  |  |  |  |  |  |  |  |  |  |  |  |  |  |  |
| Method | Level 1 | Level 2 | Average 1 | Average 2 | Difference | Standard deviation | Statistics | P value | *：P<0.05 **：P<0.01 | | | (C) | Ave. | 1/10 | Glc medium | 1/2 | 32K sup | 1/100 | 1/5 | Symbol |
| Tukey | Glc medium | 1/100 | 0.0912 | 0.0940 | 0.0028 | 0.0745 | 0.0376 | 1.0000 |  |  |  | 1/10 | 0.0943 | a | **―** | **―** | **―** | **―** | **―** | a |
|  | Glc medium | 1/10 | 0.0912 | 0.0943 | 0.0031 | 0.0745 | 0.0421 | 1.0000 |  |  |  | Glc medium | 0.0912 | a | a | **―** | **―** | **―** | **―** | a |
|  | Glc medium | 1/5 | 0.0912 | 0.0897 | 0.0015 | 0.0745 | 0.0206 | 1.0000 |  |  |  | 1/2 | 0.2867 | a | a | a | **―** | **―** | **―** | a |
|  | Glc medium | 1/2 | 0.0912 | 0.2867 | 0.1955 | 0.0745 | 2.6247 | 0.1438 |  |  |  | 32K sup | 0.2848 | a | a | a | a | **―** | **―** | a |
|  | Glc medium | 32K  supernatant | 0.0912 | 0.2848 | 0.1936 | 0.0645 | 3.0018 | 0.0734 |  |  |  | 1/100 | 0.0940 | a | a | a | a | a | **―** | a |
|  | 1/100 | 1/10 | 0.0940 | 0.0943 | 0.0003 | 0.0833 | 0.0040 | 1.0000 |  |  |  | 1/5 | 0.0897 | a | a | a | a | a | a | a |
|  | 1/100 | 1/5 | 0.0940 | 0.0897 | 0.0043 | 0.0833 | 0.0520 | 1.0000 |  |  |  | **Number of asterisks** |  | 0 | 0 | 0 | 0 | 0 | 0 |  |
|  | 1/100 | 1/2 | 0.0940 | 0.2867 | 0.1927 | 0.0833 | 2.3140 | 0.2391 |  |  |  |  |  |  |  |  |  |  |  |  |
|  | 1/100 | 32K  supernatant | 0.0940 | 0.2848 | 0.1908 | 0.0745 | 2.5620 | 0.1600 |  |  |  | 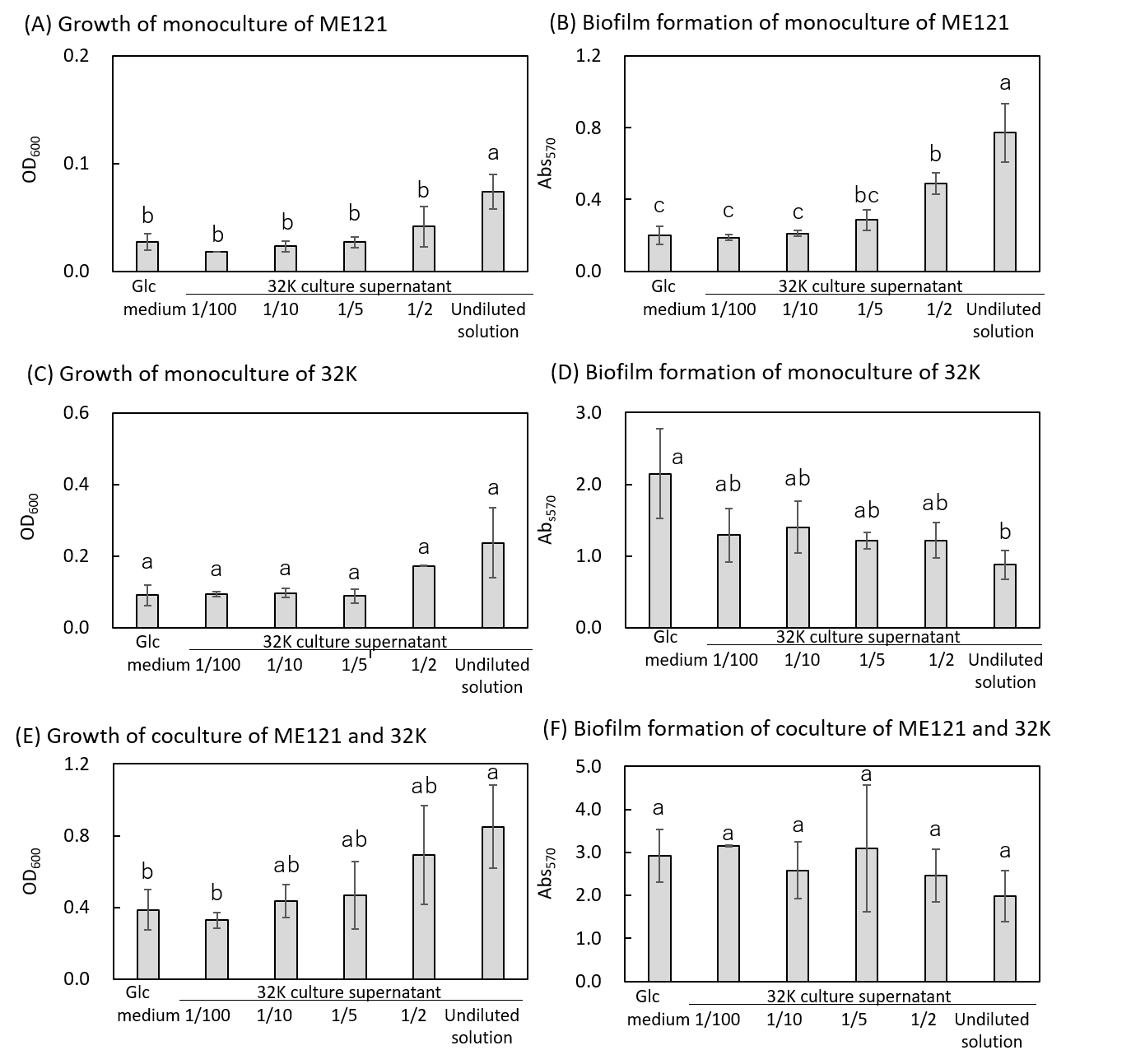 | | | | | | | | |
|  | 1/10 | 1/5 | 0.0943 | 0.0897 | 0.0047 | 0.0833 | 0.0560 | 1.0000 |  |  |  |  |  |  |  |  |  |  |  |  |
|  | 1/10 | 1/2 | 0.0943 | 0.2867 | 0.1923 | 0.0833 | 2.3100 | 0.2406 |  |  |  |  |  |  |  |  |  |  |  |  |
|  | 1/10 | 32K  supernatant | 0.0943 | 0.2848 | 0.1905 | 0.0745 | 2.5576 | 0.1612 |  |  |  |  |  |  |  |  |  |  |  |  |
|  | 1/5 | 1/2 | 0.0897 | 0.2867 | 0.1970 | 0.0833 | 2.3660 | 0.2204 |  |  |  |  |  |  |  |  |  |  |  |  |
|  | 1/5 | 32K  supernatant | 0.0897 | 0.2848 | 0.1951 | 0.0745 | 2.6202 | 0.1449 |  |  |  |  |  |  |  |  |  |  |  |  |
|  | 1/2 | 32K  supernatant | 0.2867 | 0.2848 | 0.0019 | 0.0745 | 0.0251 | 1.0000 |  |  |  |  |  |  |  |  |  |  |  |  |
|  |  |  |  |  |  |  |  |  |  |  |  |  |  |  |  |  |  |  |  |  |
| (D) Biofilm formation of monoculture of 32K | | | |  |  |  |  |  |  |  |  |  |  |  |  |  |  |  |  |  |
| Method | Level 1 | Level 2 | Average 1 | Average 2 | Difference | Standard deviation | Statistics | P value | *：P<0.05 **：P<0.01 | | | (D) | Ave. | Glc medium | 1/100 | 1/10 | 1/5 | 1/2 | 32K sup | Symbol |
| Tukey | Glc medium | 1/100 | 2.0880 | 1.2780 | 0.8100 | 0.4138 | 1.9575 | 0.3989 |  |  |  | Glc medium | 2.0880 | a | **―** | **―** | **―** | **―** | **―** | a |
|  | Glc medium | 1/10 | 2.0880 | 1.3800 | 0.7080 | 0.4138 | 1.7110 | 0.5368 |  |  |  | 1/10 | 1.3800 | a | b | **―** | **―** | **―** | **―** | ab |
|  | Glc medium | 1/5 | 2.0880 | 1.1600 | 0.9280 | 0.4138 | 2.2426 | 0.2667 |  |  |  | 1/100 | 1.2780 | a | b | b | **―** | **―** | **―** | ab |
|  | Glc medium | 1/2 | 2.0880 | 1.2167 | 0.8713 | 0.4138 | 2.1057 | 0.3259 |  |  |  | 1/2 | 1.2167 | a | b | b | b | **―** | **―** | ab |
|  | Glc medium | 32K  supernatant | 2.0880 | 0.8780 | 1.2100 | 0.3584 | 3.3765 | 0.0362 | * |  |  | 1/5 | 1.1600 | a | b | b | b | b | **―** | ab |
|  | 1/100 | 1/10 | 1.2780 | 1.3800 | 0.1020 | 0.4626 | 0.2205 | 0.9999 |  |  |  | 32K sup | 0.8780 | ＊ | b | b | b | b | b | b |
|  | 1/100 | 1/5 | 1.2780 | 1.1600 | 0.1180 | 0.4626 | 0.2551 | 0.9998 |  |  |  | **Number of asterisks** |  | 1 | 0 | 0 | 0 | 0 | 0 |  |
|  | 1/100 | 1/2 | 1.2780 | 1.2167 | 0.0613 | 0.4626 | 0.1326 | 1.0000 |  |  |  |  |  |  |  |  |  |  |  |  |
|  | 1/100 | 32K  supernatant | 1.2780 | 0.8780 | 0.4000 | 0.4138 | 0.9667 | 0.9192 |  |  |  |  |  |  |  |  |  |  |  |  |
|  | 1/10 | 1/5 | 1.3800 | 1.1600 | 0.2200 | 0.4626 | 0.4755 | 0.9962 |  |  |  |  |  |  |  |  |  |  |  |  |
|  | 1/10 | 1/2 | 1.3800 | 1.2167 | 0.1633 | 0.4626 | 0.3530 | 0.9991 |  |  |  |  |  |  |  |  |  |  |  |  |
|  | 1/10 | 32K  supernatant | 1.3800 | 0.8780 | 0.5020 | 0.4138 | 1.2131 | 0.8194 |  |  |  |  |  |  |  |  |  |  |  |  |
|  | 1/5 | 1/2 | 1.1600 | 1.2167 | 0.0567 | 0.4626 | 0.1225 | 1.0000 |  |  |  |  |  |  |  |  |  |  |  |  |
|  | 1/5 | 32K  supernatant | 1.1600 | 0.8780 | 0.2820 | 0.4138 | 0.6815 | 0.9807 |  |  |  |  |  |  |  |  |  |  |  |  |
|  | 1/2 | 32K  supernatant | 1.2167 | 0.8780 | 0.3387 | 0.4138 | 0.8184 | 0.9582 |  |  |  |  |  |  |  |  |  |  |  |  |
|  |  |  |  |  |  |  |  |  |  |  |  |  |  |  |  |  |  |  |  |  |
| (E) Growth of coculture of ME121 and 32K | | | |  |  |  |  |  |  |  |  |  |  |  |  |  |  |  |  |  |
| Method | Level 1 | Level 2 | Average 1 | Average 2 | Difference | Standard deviation | Statistics | P value | *：P<0.05 **：P<0.01 | | | (E) | Ave. | 32K | 1/2 | 1/5 | 1/10 | Glc medium | 1/100 | Symbol |
| Tukey | Glc medium | 1/100 | 0.3876 | 0.3233 | 0.0643 | 0.1493 | 0.4305 | 0.9976 |  |  |  | 32K sup | 0.8508 | a | ― | ― | ― | ― | ― | a |
|  | Glc medium | 1/10 | 0.3876 | 0.3993 | 0.0117 | 0.1493 | 0.0786 | 1.0000 |  |  |  | 1/2 | 0.6913 | a | b | ― | ― | ― | ― | ab |
|  | Glc medium | 1/5 | 0.3876 | 0.4283 | 0.0407 | 0.1493 | 0.2729 | 0.9997 |  |  |  | 1/5 | 0.4283 | a | b | b | ― | ― | ― | ab |
|  | Glc medium | 1/2 | 0.3876 | 0.6913 | 0.3037 | 0.1493 | 2.0346 | 0.3598 |  |  |  | 1/10 | 0.3993 | a | b | b | b | ― | ― | ab |
|  | Glc medium | 32K  supernatant | 0.3876 | 0.8508 | 0.4632 | 0.1293 | 3.5828 | 0.0242 | * |  |  | Glc medium | 0.3876 | * | b | b | b | b | ― | b |
|  | 1/100 | 1/10 | 0.3233 | 0.3993 | 0.0760 | 0.1669 | 0.4553 | 0.9969 |  |  |  | 1/100 | 0.3233 | * | b | b | b | b | b | b |
|  | 1/100 | 1/5 | 0.3233 | 0.4283 | 0.1050 | 0.1669 | 0.6291 | 0.9865 |  |  |  | **Number of asterisks** |  | 2 | 0 | 0 | 0 | 0 | 0 |  |
|  | 1/100 | 1/2 | 0.3233 | 0.6913 | 0.3680 | 0.1669 | 2.2048 | 0.2822 |  |  |  |  |  |  |  |  |  |  |  |  |
|  | 1/100 | 32K  supernatant | 0.3233 | 0.8508 | 0.5275 | 0.1493 | 3.5333 | 0.0267 | * |  |  | 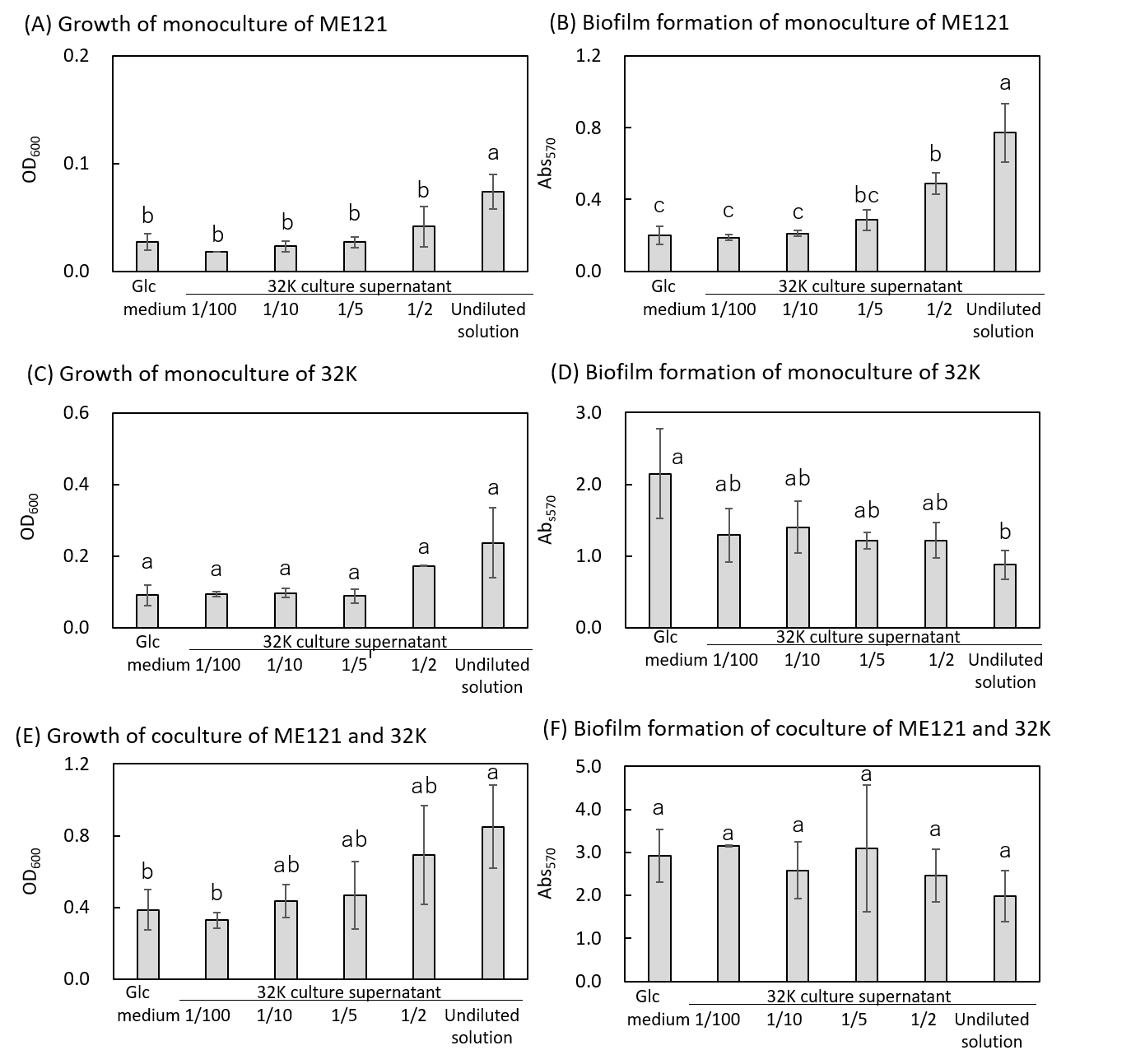 | | | | | | | | |
|  | 1/10 | 1/5 | 0.3993 | 0.4283 | 0.0290 | 0.1669 | 0.1737 | 1.0000 |  |  |  |  |  |  |  |  |  |  |  |  |
|  | 1/10 | 1/2 | 0.3993 | 0.6913 | 0.2920 | 0.1669 | 1.7495 | 0.5143 |  |  |  |  |  |  |  |  |  |  |  |  |
|  | 1/10 | 32K  supernatant | 0.3993 | 0.8508 | 0.4515 | 0.1493 | 3.0242 | 0.0705 |  |  |  |  |  |  |  |  |  |  |  |  |
|  | 1/5 | 1/2 | 0.4283 | 0.6913 | 0.2630 | 0.1669 | 1.5757 | 0.6172 |  |  |  |  |  |  |  |  |  |  |  |  |
|  | 1/5 | 32K  supernatant | 0.4283 | 0.8508 | 0.4225 | 0.1493 | 2.8299 | 0.1004 |  |  |  |  |  |  |  |  |  |  |  |  |
|  | 1/2 | 32K  supernatant | 0.6913 | 0.8508 | 0.1595 | 0.1493 | 1.0682 | 0.8832 |  |  |  |  |  |  |  |  |  |  |  |  |
|  |  |  |  |  |  |  |  |  |  |  |  |  |  |  |  |  |  |  |  |  |
| (F) Biofilm formation of coculture of ME121 and 32K | | | | |  |  |  |  |  |  |  |  |  |  |  |  |  |  |  |  |
| Method | Level 1 | Level 2 | Average 1 | Average 2 | Difference | Standard deviation | Statistics | P value | *：P<0.05 **：P<0.01 | | | (F) | Ave. | 1/2 | 1/100 | 1/5 | Glc medium | 1/10 | 32K sup | Symbol |
| Tukey | Glc medium | 1/100 | 2.9180 | 3.1007 | 0.1827 | 0.8103 | 0.2254 | 0.9999 |  |  |  | 1/2 | 3.6333 | a | **―** | **―** | **―** | **―** | **―** | a |
|  | Glc medium | 1/10 | 2.9180 | 2.7310 | 0.1870 | 0.8103 | 0.2308 | 0.9999 |  |  |  | 1/100 | 3.1070 | a | a | **―** | **―** | **―** | **―** | a |
|  | Glc medium | 1/5 | 2.9180 | 2.9533 | 0.0353 | 0.8103 | 0.0436 | 1.0000 |  |  |  | 1/5 | 2.9533 | a | a | a | **―** | **―** | **―** | a |
|  | Glc medium | 1/2 | 2.9180 | 3.6333 | 0.7153 | 0.8103 | 0.8828 | 0.9432 |  |  |  | Glc medium | 2.9180 | a | a | a | a | **―** | **―** | a |
|  | Glc medium | 32K  supernatant | 2.9180 | 2.3000 | 0.6180 | 0.7017 | 0.8807 | 0.9438 |  |  |  | 1/10 | 2.7310 | a | a | a | a | a | **―** | a |
|  | 1/100 | 1/10 | 3.1007 | 2.7310 | 0.3697 | 0.9059 | 0.4080 | 0.9982 |  |  |  | 32K sup | 2.3000 | a | a | a | a | a | a | a |
|  | 1/100 | 1/5 | 3.1007 | 2.9533 | 0.1473 | 0.9059 | 0.1626 | 1.0000 |  |  |  | **Number of asterisks** |  | 0 | 0 | 0 | 0 | 0 | 0 |  |
|  | 1/100 | 1/2 | 3.1007 | 3.6333 | 0.5327 | 0.9059 | 0.5880 | 0.9900 |  |  |  |  |  |  |  |  |  |  |  |  |
|  | 1/100 | 32K  supernatant | 3.1007 | 2.3000 | 0.8007 | 0.8103 | 0.9881 | 0.9122 |  |  |  |  |  |  |  |  |  |  |  |  |
|  | 1/10 | 1/5 | 2.7310 | 2.9533 | 0.2223 | 0.9059 | 0.2454 | 0.9998 |  |  |  |  |  |  |  |  |  |  |  |  |
|  | 1/10 | 1/2 | 2.7310 | 3.6333 | 0.9023 | 0.9059 | 0.9960 | 0.9096 |  |  |  |  |  |  |  |  |  |  |  |  |
|  | 1/10 | 32K  supernatant | 2.7310 | 2.3000 | 0.4310 | 0.8103 | 0.5319 | 0.9937 |  |  |  |  |  |  |  |  |  |  |  |  |
|  | 1/5 | 1/2 | 2.9533 | 3.6333 | 0.6800 | 0.9059 | 0.7506 | 0.9708 |  |  |  |  |  |  |  |  |  |  |  |  |
|  | 1/5 | 32K  supernatant | 2.9533 | 2.3000 | 0.6533 | 0.8103 | 0.8063 | 0.9607 |  |  |  |  |  |  |  |  |  |  |  |  |
|  | 1/2 | 32K  supernatant | 3.6333 | 2.3000 | 1.3333 | 0.8103 | 1.6455 | 0.5756 |  |  |  |  |  |  |  |  |  |  |  |  |

**Table S4.** Tukey test data for post hoc analysis of the results in Figure 4.

| **Figure 4** | | | | | | | | | | | | | | | | | | | | |
| --- | --- | --- | --- | --- | --- | --- | --- | --- | --- | --- | --- | --- | --- | --- | --- | --- | --- | --- | --- | --- |
| Multiple comparison analysis testing | | |  |  |  |  |  |  |  |  |  |  |  |  |  |  |  |  |  |  |
| Figure 4A. Biofilm formation in monocultures of strains ME121 and 32K, and coculture of strains ME121 and 32K when the initial inoculum of strain ME121 was changed. | | | | | | | | | | | | | | | | | | | | |
| Method | Level 1 | Level 2 | Ave. 1 | Ave. 2 | Difference | Standard deviation | Statistics | P value | *：P<0.05 **：P<0.01 | |  |  |  |  |  |  |  |  |  |  |
| Tukey | Glc medium | ME121=0.001 | 0.0630 | 0.1122 | 0.0492 | 0.0739 | 0.6655 | 0.9974 |  | Figure 4A | Ave. | ME121+32K=0.001 | ME121+32K=0.01 | ME121+32K=0.1 | 32K=0.01 | ME121=0.001 | ME121=0.01 | ME121=0.1 | Glc medium | Symbol |
|  | Glc medium | 32K=0.01 | 0.0630 | 0.8980 | 0.8350 | 0.0739 | 11.2947 | P < 0.001 | ** | ME121+32K=0.001 | 1.4560 | a | **―** | **―** | **―** | **―** | **―** | **―** | **―** | a |
|  | Glc medium | ME121+32K=0.001 | 0.0630 | 1.4560 | 1.3930 | 0.0739 | 18.8425 | P < 0.001 | ** | ME121+32K=0.01 | 1.3360 | a | ｂ | **―** | **―** | **―** | **―** | **―** | **―** | ab |
|  | Glc medium | ME121=0.01 | 0.0630 | 0.0904 | 0.0274 | 0.0739 | 0.3706 | 0.9999 |  | ME121+32K=0.1 | 1.1360 | ＊ | ｂ | ｃ | **―** | **―** | **―** | **―** | **―** | bc |
|  | Glc medium | ME121+32K=0.01 | 0.0630 | 1.3360 | 1.2730 | 0.0739 | 17.2193 | P < 0.001 | ** | 32K=0.01 | 0.8980 | ＊ | ＊ | ｃ | c | **―** | **―** | **―** | **―** | c |
|  | Glc medium | ME121=0.1 | 0.0630 | 0.0904 | 0.0274 | 0.0739 | 0.3706 | 0.9999 |  | ME121=0.001 | 0.1122 | ＊ | ＊ | ＊ | ＊ | d | **―** | **―** | **―** | d |
|  | Glc medium | ME121+32K=0.1 | 0.0630 | 1.1360 | 1.0730 | 0.0739 | 14.5140 | P < 0.001 | ** | ME121=0.01 | 0.0904 | ＊ | ＊ | ＊ | ＊ | d | d | **―** | **―** | d |
|  | ME121=0.001 | 32K=0.01 | 0.1122 | 0.8980 | 0.7858 | 0.0739 | 10.6292 | P < 0.001 | ** | ME121=0.1 | 0.0904 | ＊ | ＊ | ＊ | ＊ | d | d | d | **―** | d |
|  | ME121=0.001 | ME121+32K=0.001 | 0.1122 | 1.4560 | 1.3438 | 0.0739 | 18.1770 | P < 0.001 | ** | Glc medium | 0.0630 | ＊ | ＊ | ＊ | ＊ | d | d | d | d | d |
|  | ME121=0.001 | ME121=0.01 | 0.1122 | 0.0904 | 0.0218 | 0.0739 | 0.2949 | 1.0000 |  | **Number of asterisks** |  | 6 | 5 | 4 | 4 | 0 | 0 | 0 | 0 |  |
|  | ME121=0.001 | ME121+32K=0.01 | 0.1122 | 1.3360 | 1.2238 | 0.0739 | 16.5538 | P < 0.001 | ** | The symbols with a, b, c and d are assigned from the one with the largest number of asterisks. | | | | | | | | | | |
|  | ME121=0.001 | ME121=0.1 | 0.1122 | 0.0904 | 0.0218 | 0.0739 | 0.2949 | 1.0000 |  |  |  | \|  \| \| --- \| |  |  |  |  |  |  |  |  |
|  | ME121=0.001 | ME121+32K=0.1 | 0.1122 | 1.1360 | 1.0238 | 0.0739 | 13.8485 | P < 0.001 | ** |  |  |  |  |  |  |  |  |  |  |  |
|  | 32K=0.01 | ME121+32K=0.001 | 0.8980 | 1.4560 | 0.5580 | 0.0739 | 7.5478 | P < 0.001 | ** |  |  |  |  |  |  |  |  |  |  |  |
|  | 32K=0.01 | ME121=0.01 | 0.8980 | 0.0904 | 0.8076 | 0.0739 | 10.9241 | P < 0.001 | ** | 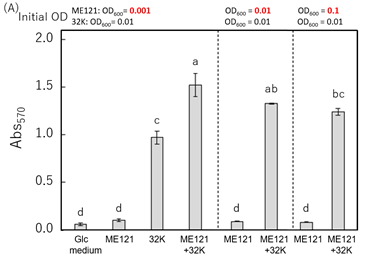 | | | | | | | | | | |
|  | 32K=0.01 | ME121+32K=0.01 | 0.8980 | 1.3360 | 0.4380 | 0.0739 | 5.9246 | P < 0.001 | ** |  |  |  |  |  |  |  |  |  |  |  |
|  | 32K=0.01 | ME121=0.1 | 0.8980 | 0.0904 | 0.8076 | 0.0739 | 10.9241 | P < 0.001 | ** |  |  |  |  |  |  |  |  |  |  |  |
|  | 32K=0.01 | ME121+32K=0.1 | 0.8980 | 1.1360 | 0.2380 | 0.0739 | 3.2193 | 0.0524 |  |  |  |  |  |  |  |  |  |  |  |  |
|  | ME121+32K=0.001 | ME121=0.01 | 1.4560 | 0.0904 | 1.3656 | 0.0739 | 18.4719 | P < 0.001 | ** |  |  |  |  |  |  |  |  |  |  |  |
|  | ME121+32K=0.001 | ME121+32K=0.01 | 1.4560 | 1.3360 | 0.1200 | 0.0739 | 1.6232 | 0.7333 |  |  |  |  |  |  |  |  |  |  |  |  |
|  | ME121+32K=0.001 | ME121=0.1 | 1.4560 | 0.0904 | 1.3656 | 0.0739 | 18.4719 | P < 0.001 | ** |  |  |  |  |  |  |  |  |  |  |  |
|  | ME121+32K=0.001 | ME121+32K=0.1 | 1.4560 | 1.1360 | 0.3200 | 0.0739 | 4.3285 | 0.0031 | ** |  |  |  |  |  |  |  |  |  |  |  |
|  | ME121=0.01 | ME121+32K=0.01 | 0.0904 | 1.3360 | 1.2456 | 0.0739 | 16.8487 | P < 0.001 | ** |  |  |  |  |  |  |  |  |  |  |  |
|  | ME121=0.01 | ME121=0.1 | 0.0904 | 0.0904 | 0.0000 | 0.0739 | 0.0000 | 1.0000 |  |  |  |  |  |  |  |  |  |  |  |  |
|  | ME121=0.01 | ME121+32K=0.1 | 0.0904 | 1.1360 | 1.0456 | 0.0739 | 14.1434 | P < 0.001 | ** |  |  |  |  |  |  |  |  |  |  |  |
|  | ME121+32K=0.01 | ME121=0.1 | 1.3360 | 0.0904 | 1.2456 | 0.0739 | 16.8487 | P < 0.001 | ** |  |  |  |  |  |  |  |  |  |  |  |
|  | ME121+32K=0.01 | ME121+32K=0.1 | 1.3360 | 1.1360 | 0.2000 | 0.0739 | 2.7053 | 0.1577 |  |  |  |  |  |  |  |  |  |  |  |  |
|  | ME121=0.1 | ME121+32K=0.1 | 0.0904 | 1.1360 | 1.0456 | 0.0739 | 14.1434 | P < 0.001 | ** |  |  |  |  |  |  |  |  |  |  |  |
|  |  |  |  |  |  |  |  |  |  |  |  |  |  |  |  |  |  |  |  |  |
| Figure 4B. Biofilm formation in monocultures of strains ME121 and 32K, and coculture of strains ME121 and 32K when the initial inoculum of strain 32K was changed. | | | | | | | | | | | | | | | | | | | | |
| Method | Level 1 | Level 2 | Ave. 1 | Ave. 2 | Difference | Standard deviation | Statistics | P value | *：P<0.05 **：P<0.01 | |  |  |  |  |  |  |  |  |  |  |
| Tukey | Glc medium | ME121=0.001 | 0.0543 | 0.0895 | 0.0353 | 0.1223 | 0.2883 | 1.0000 |  |  |  |  |  |  |  |  |  |  |  |  |
|  | Glc medium | 32K=0.001 | 0.0543 | 0.2900 | 0.2358 | 0.1223 | 1.9282 | 0.5463 |  | Figure 4B | Ave. | ME121+32K=0.01 | ME121+32K=0.1 | ME121+32K=0.001 | 32K=0.01 | 32K=0.1 | 32K=0.001 | ME121=0.01 | Glc medium | Symbol |
|  | Glc medium | ME121+32K=0.001 | 0.0543 | 1.5850 | 1.5308 | 0.1223 | 12.5197 | P < 0.001 | ** | ME121+32K=0.01 | 1.7500 | a | **―** | **―** | **―** | **―** | **―** | **―** | **―** | a |
|  | Glc medium | 32K=0.01 | 0.0543 | 1.2200 | 1.1658 | 0.1223 | 9.5345 | P < 0.001 | ** | ME121+32K=0.1 | 1.6875 | a | a | **―** | **―** | **―** | **―** | **―** | **―** | a |
|  | Glc medium | ME121+32K=0.01 | 0.0543 | 1.7500 | 1.6958 | 0.1223 | 13.8693 | P < 0.001 | ** | ME121+32K=0.001 | 1.5850 | a | a | b | **―** | **―** | **―** | **―** | **―** | ab |
|  | Glc medium | 32K=0.1 | 0.0543 | 1.1000 | 1.0458 | 0.1223 | 8.5530 | P < 0.001 | ** | 32K=0.01 | 1.2200 | ＊ | ＊ | b | c | **―** | **―** | **―** | **―** | bc |
|  | Glc medium | ME121+32K=0.1 | 0.0543 | 1.6875 | 1.6333 | 0.1223 | 13.3581 | P < 0.001 | ** | 32K=0.1 | 1.1000 | ＊ | ＊ | ＊ | c | c | **―** | **―** | **―** | c |
|  | ME121=0.01 | 32K=0.001 | 0.0895 | 0.2900 | 0.2005 | 0.1223 | 1.6399 | 0.7229 |  | 32K=0.001 | 0.2900 | ＊ | ＊ | ＊ | ＊ | ＊ | d | **―** | **―** | d |
|  | ME121=0.01 | ME121+32K=0.001 | 0.0895 | 1.5850 | 1.4955 | 0.1223 | 12.2314 | P < 0.001 | ** | ME121=0.01 | 0.0895 | ＊ | ＊ | ＊ | ＊ | ＊ | d | d | **―** | d |
|  | ME121=0.01 | 32K=0.01 | 0.0895 | 1.2200 | 1.1305 | 0.1223 | 9.2462 | P < 0.001 | ** | Glc medium | 0.0543 | ＊ | ＊ | ＊ | ＊ | ＊ | d | d | d | d |
|  | ME121=0.01 | ME121+32K=0.01 | 0.0895 | 1.7500 | 1.6605 | 0.1223 | 13.5810 | P < 0.001 | ** | **Number of asterisks** |  | 5 | 5 | 4 | 3 | 3 | 0 | 0 | 0 |  |
|  | ME121=0.01 | 32K=0.1 | 0.0895 | 1.1000 | 1.0105 | 0.1223 | 8.2647 | P < 0.001 | ** | The symbols with a, b, c and d are assigned from the one with the largest number of asterisks. | | | | | | | | | | |
|  | ME121=0.01 | ME121+32K=0.1 | 0.0895 | 1.6875 | 1.5980 | 0.1223 | 13.0698 | P < 0.001 | ** |  |  |  |  |  |  |  |  |  |  |  |
|  | 32K=0.001 | ME121+32K=0.001 | 0.2900 | 1.5850 | 1.2950 | 0.1223 | 10.5916 | P < 0.001 | ** | 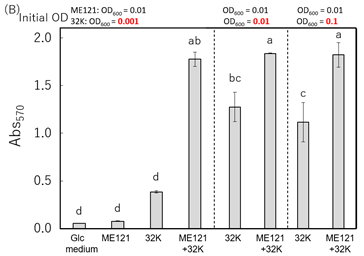 | | | | | | | | | | |
|  | 32K=0.001 | 32K=0.01 | 0.2900 | 1.2200 | 0.9300 | 0.1223 | 7.6063 | P < 0.001 | ** |  |  |  |  |  |  |  |  |  |  |  |
|  | 32K=0.001 | ME121+32K=0.01 | 0.2900 | 1.7500 | 1.4600 | 0.1223 | 11.9411 | P < 0.001 | ** |  |  |  |  |  |  |  |  |  |  |  |
|  | 32K=0.001 | 32K=0.1 | 0.2900 | 1.1000 | 0.8100 | 0.1223 | 6.6249 | P < 0.001 | ** |  |  |  |  |  |  |  |  |  |  |  |
|  | 32K=0.001 | ME121+32K=0.1 | 0.2900 | 1.6875 | 1.3975 | 0.1223 | 11.4299 | P < 0.001 | ** |  |  |  |  |  |  |  |  |  |  |  |
|  | ME121+32K=0.001 | 32K=0.01 | 1.5850 | 1.2200 | 0.3650 | 0.1223 | 2.9853 | 0.0988 |  |  |  |  |  |  |  |  |  |  |  |  |
|  | ME121+32K=0.001 | ME121+32K=0.01 | 1.5850 | 1.7500 | 0.1650 | 0.1223 | 1.3495 | 0.8704 |  |  |  |  |  |  |  |  |  |  |  |  |
|  | ME121+32K=0.001 | 32K=0.1 | 1.5850 | 1.1000 | 0.4850 | 0.1223 | 3.9667 | 0.0113 | * |  |  |  |  |  |  |  |  |  |  |  |
|  | ME121+32K=0.001 | ME121+32K=0.1 | 1.5850 | 1.6875 | 0.1025 | 0.1223 | 0.8383 | 0.9888 |  |  |  |  |  |  |  |  |  |  |  |  |
|  | 32K=0.01 | ME121+32K=0.01 | 1.2200 | 1.7500 | 0.5300 | 0.1223 | 4.3348 | 0.0047 | ** |  |  |  |  |  |  |  |  |  |  |  |
|  | 32K=0.01 | 32K=0.1 | 1.2200 | 1.1000 | 0.1200 | 0.1223 | 0.9815 | 0.9729 |  |  |  |  |  |  |  |  |  |  |  |  |
|  | 32K=0.01 | ME121+32K=0.1 | 1.2200 | 1.6875 | 0.4675 | 0.1223 | 3.8236 | 0.0158 | * |  |  |  |  |  |  |  |  |  |  |  |
|  | ME121+32K=0.01 | 32K=0.1 | 1.7500 | 1.1000 | 0.6500 | 0.1223 | 5.3162 | P < 0.001 | ** |  |  |  |  |  |  |  |  |  |  |  |
|  | ME121+32K=0.01 | ME121+32K=0.1 | 1.7500 | 1.6875 | 0.0625 | 0.1223 | 0.5112 | 0.9995 |  |  |  |  |  |  |  |  |  |  |  |  |
|  | 32K=0.1 | ME121+32K=0.1 | 1.1000 | 1.6875 | 0.5875 | 0.1223 | 4.8051 | 0.0015 | ** |  |  |  |  |  |  |  |  |  |  |  |


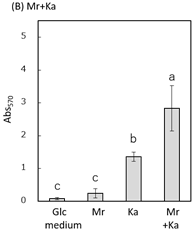

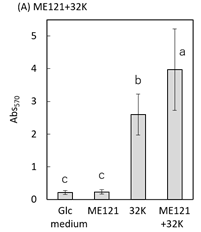
**Table S5.** Tukey test data for post hoc analysis of the results in Figure 5.

| **Figure 5** | | | | | | | | | | | | | | | | | | |
| --- | --- | --- | --- | --- | --- | --- | --- | --- | --- | --- | --- | --- | --- | --- | --- | --- | --- | --- |
| Multiple comparison analysis testing | | | | | | | | | | | | | | | | | | |
| (A) ME121 + 32K | |  |  |  |  |  |  |  |  |  |  |  |  |  |  |  |  |  |
| Method | Level 1 | Level 2 | Ave. 1 | Ave. 2 | Difference | Standard deviation | Statistics | P value | *：P<0.05 **：P<0.01 | | | (A) ME121 + 32K | Ave. | ME121+32K | 32K | ME121 | Glc medium | Symbol |
| Tukey | Glc medium | ME121 | 0.2143 | 0.2297 | 0.0154 | 0.2641 | 0.0583 | 0.9999 |  |  |  | ME121+32K | 3.9767 | a | **―** | **―** | **―** | a |
|  | Glc medium | 32K | 0.2143 | 2.5993 | 2.3850 | 0.2641 | 9.0301 | P < 0.001 | ** |  |  | 32K | 2.5993 | * | b | **―** | **―** | b |
|  | Glc medium | ME121+32K | 0.2143 | 3.9767 | 3.7623 | 0.2641 | 14.2449 | P < 0.001 | ** |  |  | ME121 | 0.2297 | * | * | c | **―** | c |
|  | ME121 | 32K | 0.2297 | 2.5993 | 2.3696 | 0.2641 | 8.9718 | P < 0.001 | ** |  |  | Glc medium | 0.2143 | * | * | c | c | c |
|  | ME121 | ME121+32K | 0.2297 | 3.9767 | 3.7469 | 0.2641 | 14.1866 | P < 0.001 | ** |  |  | **Number of asterisks** |  | 3 | 2 | 0 | 0 |  |
|  | 32K | ME121+32K | 2.5993 | 3.9767 | 1.3773 | 0.2641 | 5.2149 | P < 0.001 | ** |  |  | The symbols with a, b, c and d are assigned from the one with the largest number of asterisks. | | | | | | |
|  |  |  |  |  |  |  |  |  |  |  |  |  |  |  |  |  |  |  |
| (B) Mr + Ka | |  |  |  |  |  |  |  |  |  |  |  |  |  |  |  |  |  |
| Method | Level 1 | Level 2 | Ave. 1 | Ave. 2 | Difference | Standard deviation | Statistics | P value | *：P<0.05 **：P<0.01 | | | (B) Mr + Ka | Ave. | Mr+Ka | Ka | Mr | Glc medium | Symbol |
| Tukey | Glc medium | Mr | 0.0760 | 0.2432 | 0.1672 | 0.2556 | 0.6541 | 0.9126 |  |  |  | Mr+Ka | 2.8300 | a | **―** | **―** | **―** | a |
|  | Glc medium | Ka | 0.0760 | 1.3620 | 1.2860 | 0.2556 | 5.0313 | P < 0.001 | ** |  |  | Ka | 1.3620 | ＊ | b | **―** | **―** | b |
|  | Glc medium | Mr+Ka | 0.0760 | 2.8320 | 2.7560 | 0.2556 | 10.7825 | P < 0.001 | ** |  |  | Mr | 0.2432 | ＊ | ＊ | c | **―** | c |
|  | Mr | Ka | 0.2432 | 1.3620 | 1.1188 | 0.2556 | 4.3771 | 0.0024 | ** |  |  | Glc medium | 0.0760 | ＊ | ＊ | c | c | c |
|  | Mr | Mr+Ka | 0.2432 | 2.8320 | 2.5888 | 0.2556 | 10.1283 | P < 0.001 | ** |  |  | **Number of asterisks** |  | 3 | 2 | 0 | 0 |  |
|  | Ka | Mr+Ka | 1.3620 | 2.8320 | 1.4700 | 0.2556 | 5.7512 | P < 0.001 | ** |  |  |  |  |  |  |  |  |  |
|  |  |  |  |  |  |  |  |  |  |  |  |  |  |  |  |  |  |  |
| (C) ME121 + Ka | |  |  |  |  |  |  |  |  |  |  |  |  |  |  |  |  |  |
| Method | Level 1 | Level 2 | Ave. 1 | Ave. 2 | Difference | Standard deviation | Statistics | P value | *：P<0.05 **：P<0.01 | | | (C) ME121 + Ka | Ave. | ME121+Ka | Ka | ME121 | Glc medium | Symbol |
| Tukey | Glc medium | ME121 | 0.0760 | 0.1078 | 0.0318 | 0.1067 | 0.2980 | 0.9904 |  |  |  | ME121+Ka | 3.2400 | a | **―** | **―** | **―** | a |
|  | Glc medium | Ka | 0.0760 | 1.3620 | 1.2860 | 0.1067 | 12.0524 | P < 0.001 | ** |  |  | Ka | 1.3620 | * | b | **―** | **―** | b |
|  | Glc medium | ME121+Ka | 0.0760 | 3.2400 | 3.1640 | 0.1067 | 29.6529 | P < 0.001 | ** |  |  | ME121 | 0.1078 | * | * | c | **―** | c |
|  | ME121 | Ka | 0.1078 | 1.3620 | 1.2542 | 0.1067 | 11.7543 | P < 0.001 | ** |  |  | Glc medium | 0.0760 | * | * | c | c | c |
|  | ME121 | ME121+Ka | 0.1078 | 3.2400 | 3.1322 | 0.1067 | 29.3549 | P < 0.001 | ** |  |  | **Number of asterisks** |  | 3 | 2 | 0 | 0 |  |
|  | Ka | ME121+Ka | 1.3620 | 3.2400 | 1.8780 | 0.1067 | 17.6006 | P < 0.001 | ** |  |  |  |  |  |  |  |  |  |
|  |  |  |  |  |  |  |  |  |  |  |  |  |  |  |  |  |  |  |
| (D) Mr +32K | |  |  |  |  |  |  |  |  |  |  |  |  |  |  |  |  |  |
| Method | Level 1 | Level 2 | Ave. 1 | Ave. 2 | Difference | Standard deviation | Statistics | P value | 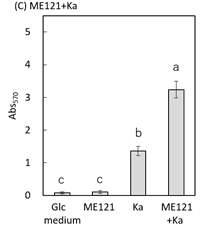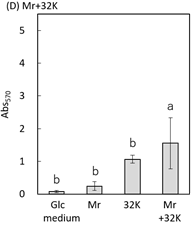*：P<0.05 **：P<0.01 | | | (D) Mr +32K | 平均 | Mr+32K | 32K | Mr | Glc medium | Symbol |
| Tukey | Glc medium | Mr | 0.0760 | 0.2432 | 0.1672 | 0.2835 | 0.5898 | 0.9337 |  |  |  | Mr+32K | 1.5520 | a | **―** | **―** | **―** | a |
|  | Glc medium | 32K | 0.0760 | 0.7260 | 0.6500 | 0.2835 | 2.2929 | 0.1413 |  |  |  | 32K | 0.7260 | ＊ | b | **―** | **―** | b |
|  | Glc medium | Mr+32K | 0.0760 | 1.5520 | 1.4760 | 0.2835 | 5.2067 | P < 0.001 | ** |  |  | Mr | 0.2432 | ＊ | b | b | **―** | b |
|  | Mr | 32K | 0.2432 | 0.7260 | 0.4828 | 0.2835 | 1.7031 | 0.3542 |  |  |  | Glc medium | 0.0760 | ＊ | b | b | b | b |
|  | Mr | Mr+32K | 0.2432 | 1.5520 | 1.3088 | 0.2835 | 4.6169 | 0.0015 | ** |  |  | **Number of asterisks** |  | 3 | 0 | 0 | 0 |  |
|  | 32K | Mr+32K | 0.7260 | 1.5520 | 0.8260 | 0.2835 | 2.9138 | 0.0452 | * |  |  |  |  |  |  |  |  |  |


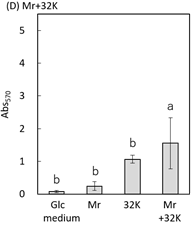

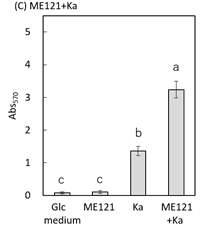


**Table S6.** Tukey test data for post hoc analysis of the results in Figure 6.

| Figure 6 | | | | | | | | | | | | | | | |
| --- | --- | --- | --- | --- | --- | --- | --- | --- | --- | --- | --- | --- | --- | --- | --- |
| Multiple comparison analysis testing | | |  |  |  |  |  |  |  |  |  |  |  |  |  |
| ME121 |  |  |  |  |  |  |  |  |  |  |  |  |  |  |  |
| Method | Level 1 | Level 2 | Ave. 1 | Ave. 2 | Difference | Standard deviation | Statistics | P value | *：P<0.05 **：P<0.01 |  | Ave. | ME121-32K sup | ME121-KA sup | ME121-Glc medium | Symbol |
| Tukey | ME121-Glc medium | ME121-32K sup. | 37.3704 | 50.7324 | 13.3620 | 0.6845 | 19.5201 | P < 0.001 | ** | ME121-32K sup. | 50.7324 | a | **―** | **―** | a |
|  | ME121-Glc medium | ME121-KA sup. | 37.3704 | 48.9878 | 11.6174 | 0.6845 | 16.9714 | P < 0.001 | ** | ME121-KA sup. | 48.9878 | ＊ | b | **―** | b |
|  | ME121-32K sup. | ME121-KA sup. | 50.7324 | 48.9878 | 1.7446 | 0.6845 | 2.5486 | 0.0305 | * | ME121-Glc medium | 37.3704 | ＊ | ＊ | c | c |
|  |  |  |  |  |  |  |  |  |  | **Number of asterisks** |  | 2 | 1 | 0 |  |
|  |  |  |  |  |  |  |  |  |  | 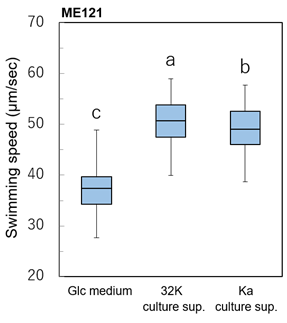 | | | | | |
|  |  |  |  |  |  |  |  |  |  |  |  |  |  |  |  |
|  |  |  |  |  |  |  |  |  |  |  |  |  |  |  |  |
|  |  |  |  |  |  |  |  |  |  |  |  |  |  |  |  |
|  |  |  |  |  |  |  |  |  |  |  |  |  |  |  |  |
|  |  |  |  |  |  |  |  |  |  |  |  |  |  |  |  |
|  |  |  |  |  |  |  |  |  |  |  |  |  |  |  |  |
|  |  |  |  |  |  |  |  |  |  |  |  |  |  |  |  |
| *M. radiotolerans* | |  |  |  |  |  |  |  |  |  |  |  |  |  |  |
| Method | Level 1 | Level 2 | Ave. 1 | Ave. 2 | Difference | Standard deviation | Statistics | P value | *：P<0.05 **：P<0.01 |  | Ave. | Mrad-KA sup | Mrad-32K sup | Mrad-Glc medium | Symbol |
| Tukey | Mrad-Glc medium | Mrad-32K sup. | 37.4936 | 42.3888 | 4.8952 | 0.6926 | 7.0682 | P < 0.001 | ** | Mrad-KA sup. | 47.2978 | a | **―** | **―** | a |
|  | Mrad-Glc medium | Mrad-KA sup. | 37.4936 | 47.2978 | 9.8042 | 0.6926 | 14.1564 | P < 0.001 | ** | Mrad-32K sup. | 42.3888 | ＊ | b | **―** | b |
|  | Mrad-32K sup. | Mrad-KA sup. | 42.3888 | 47.2978 | 4.9090 | 0.6926 | 7.0882 | P < 0.001 | ** | Mrad-Glc medium | 37.4936 | ＊ | ＊ | c | c |
|  |  |  |  |  |  |  |  |  |  | **Number of asterisks** |  | 2 | 1 | 0 |  |
| 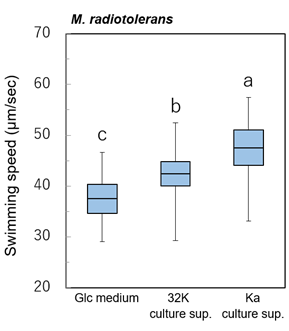 | | | | | | | | | | | | | | | |
