# Supplementary material for: Metabolites Produced by Kaistia sp. 32K Promote Biofilm Formation in Coculture with Methylobacterium sp. ME121
Source: Biology (Basel). 2020 Sep 13;9(9):287. doi: 10.3390/biology9090287 (PMC7563137; doi:10.3390/biology9090287)
Supplement: Supplementary file 1 [file biology-09-00287-s001.zip › biology-902103-supplementary-final/supplimentary files/supplimental videoü@final.pptx]

## Slide 1
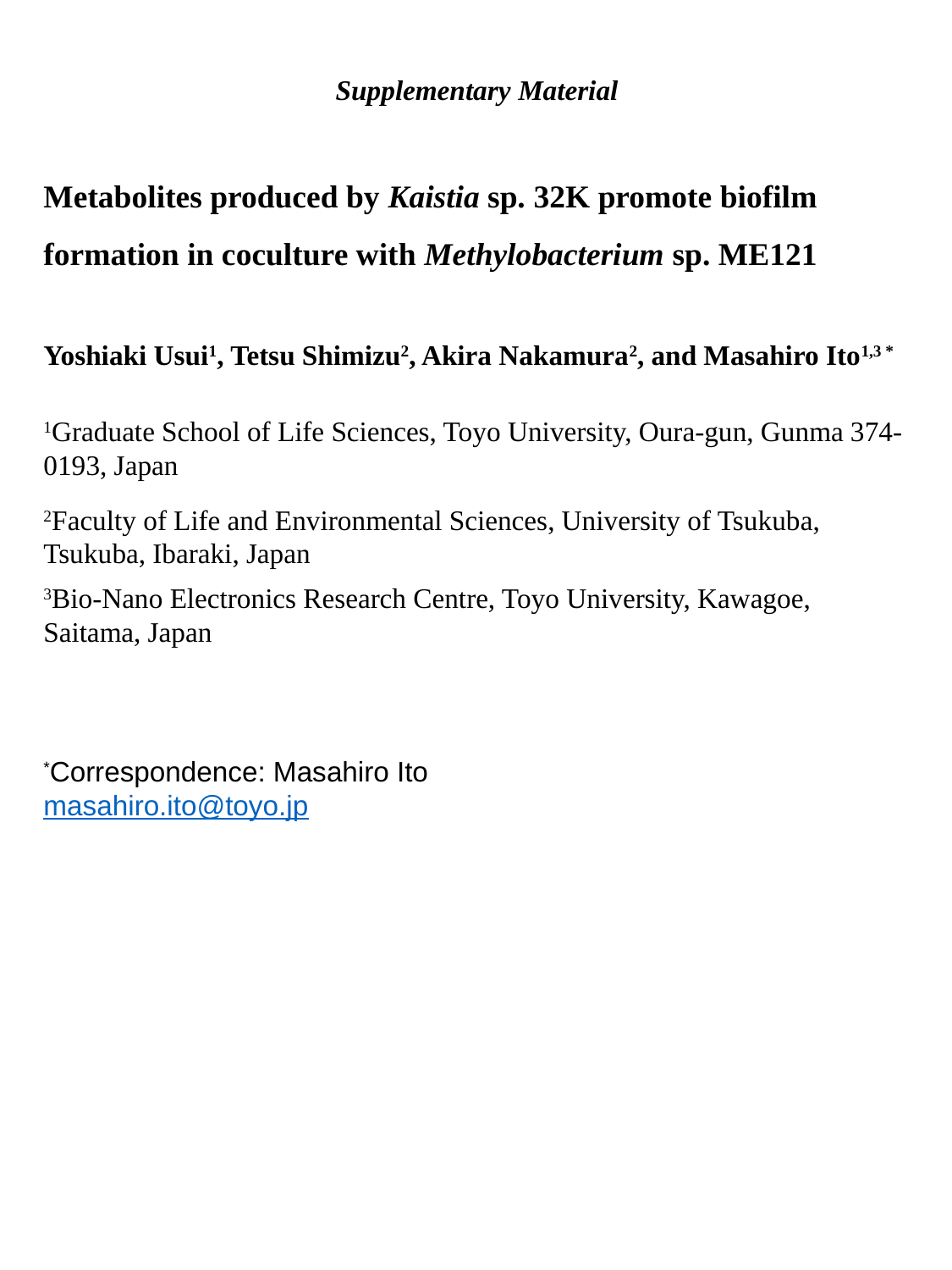

Supplementary Material
Metabolites produced by Kaistia sp. 32K promote biofilm formation in coculture with Methylobacterium sp. ME121
Yoshiaki Usui1, Tetsu Shimizu2, Akira Nakamura2, and Masahiro Ito1,3 *
1Graduate School of Life Sciences, Toyo University, Oura-gun, Gunma 374-0193, Japan
2Faculty of Life and Environmental Sciences, University of Tsukuba, Tsukuba, Ibaraki, Japan
3Bio-Nano Electronics Research Centre, Toyo University, Kawagoe, Saitama, Japan
*Correspondence: Masahiro Ito
masahiro.ito@toyo.jp

## Slide 2
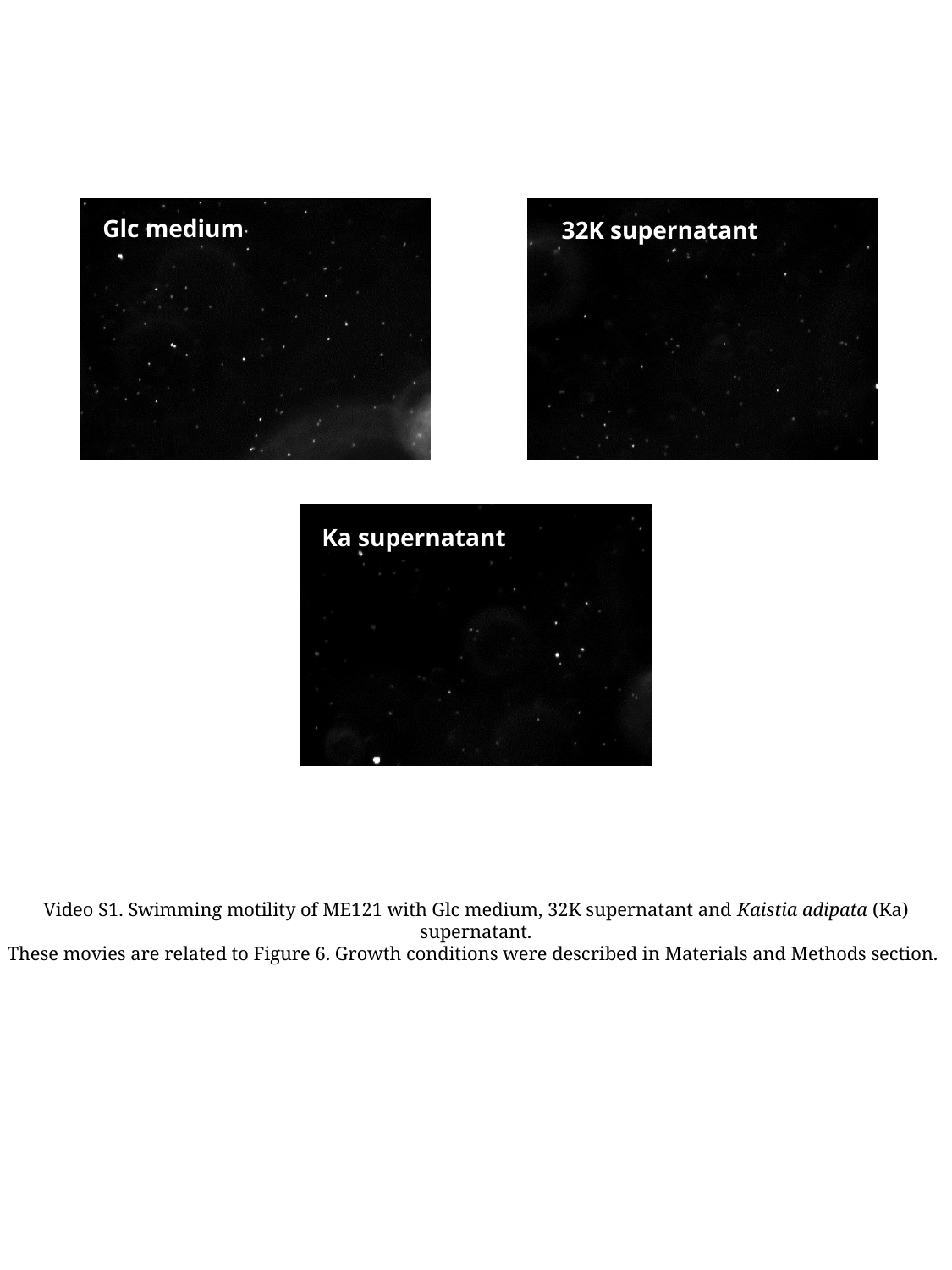

Glc medium
32K supernatant
Ka supernatant
Video S1. Swimming motility of ME121 with Glc medium, 32K supernatant and Kaistia adipata (Ka) supernatant.
These movies are related to Figure 6. Growth conditions were described in Materials and Methods section.

## Slide 3
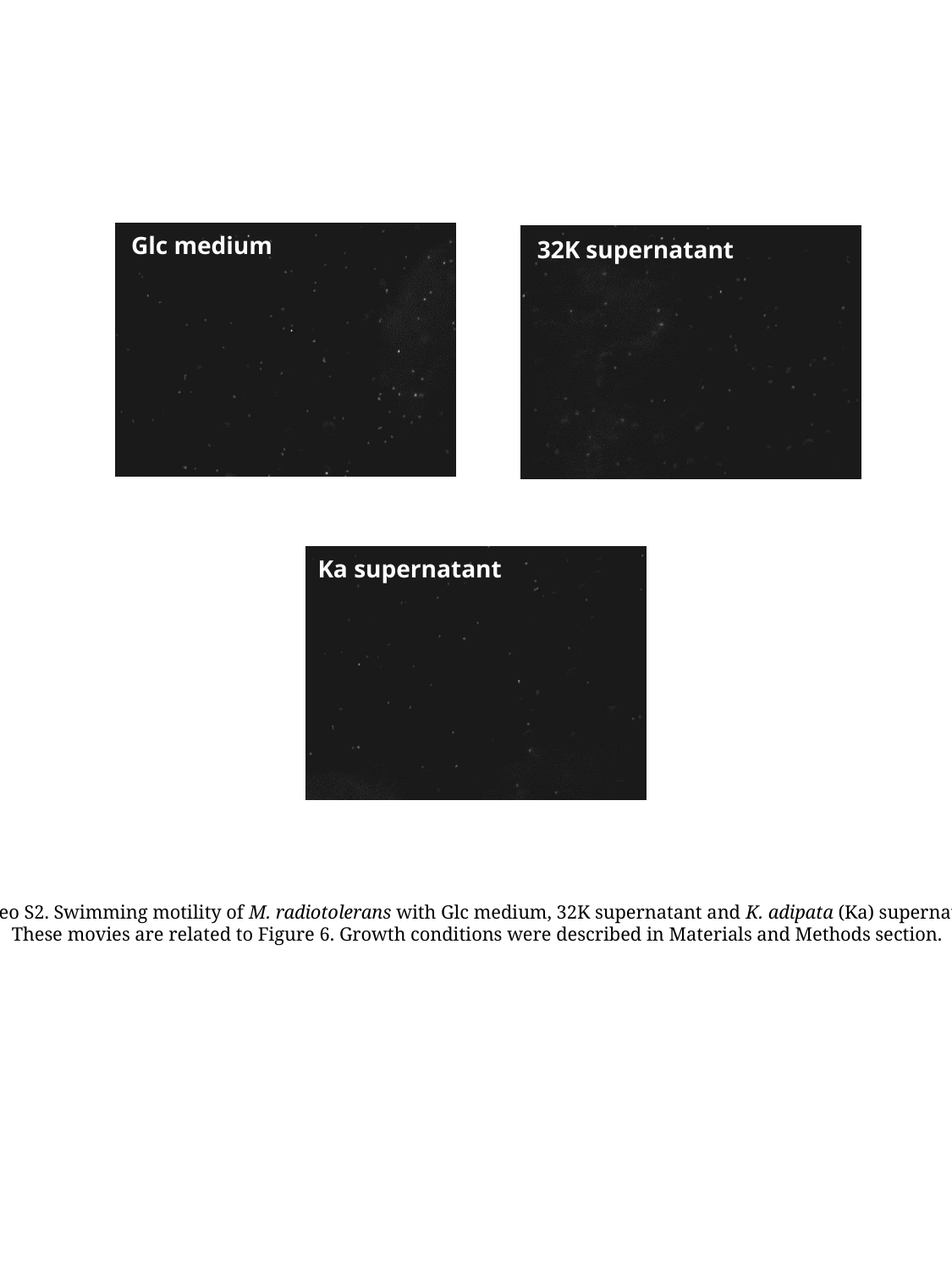

Glc medium
32K supernatant
Ka supernatant
Video S2. Swimming motility of M. radiotolerans with Glc medium, 32K supernatant and K. adipata (Ka) supernatant.
These movies are related to Figure 6. Growth conditions were described in Materials and Methods section.
